# Combined noncanonical NF-**κ**B agonism and targeted BET bromodomain inhibition reverse HIV latency ex vivo

**DOI:** 10.1172/JCI157281

**Published:** 2022-04-15

**Authors:** Shane D. Falcinelli, Jackson J. Peterson, Anne-Marie W. Turner, David Irlbeck, Jenna Read, Samuel L.M. Raines, Katherine S. James, Cameron Sutton, Anthony Sanchez, Ann Emery, Gavin Sampey, Robert Ferris, Brigitte Allard, Simon Ghofrani, Jennifer L. Kirchherr, Caroline Baker, JoAnn D. Kuruc, Cynthia L. Gay, Lindsey I. James, Guoxin Wu, Paul Zuck, Inmaculada Rioja, Rebecca C. Furze, Rab K. Prinjha, Bonnie J. Howell, Ronald Swanstrom, Edward P. Browne, Brian D. Strahl, Richard M. Dunham, Nancie M. Archin, David M. Margolis

**Affiliations:** 1 UNC HIV Cure Center, University of North Carolina (UNC), Chapel Hill, North Carolina, USA.; 2 Department of Microbiology and Immunology, UNC School of Medicine, Chapel Hill, North Carolina, USA.; 3 Division of Infectious Diseases, Department of Medicine, UNC, Chapel Hill, North Carolina, USA.; 4 HIV Drug Discovery, ViiV Healthcare, Research Triangle Park, North Carolina, USA.; 5 Department of Biochemistry and Biophysics, UNC School of Medicine, Chapel Hill, North Carolina, USA.; 6 Division of Chemical Biology and Medicinal Chemistry, UNC Eshelman School of Pharmacy, Chapel Hill, North Carolina, USA.; 7 Department of Infectious Disease, Merck & Co. Inc., Kenilworth, New Jersey, USA.; 8 Immuno-Epigenetics, Immunology Research Unit, GSK Medicines Research Centre, Stevenage, United Kingdom.

**Keywords:** AIDS/HIV, Infectious disease, RNA processing, T cells, Transcription

## Abstract

Latency reversal strategies for HIV cure using inhibitor of apoptosis protein (IAP) antagonists (IAPi) induce unprecedented levels of latent reservoir expression without immunotoxicity during suppressive antiretroviral therapy (ART). However, full targeting of the reservoir may require combinatorial approaches. A Jurkat latency model screen for IAPi combination partners demonstrated synergistic latency reversal with bromodomain (BD) and extraterminal domain protein inhibitors (BETi). Mechanistic investigations using CRISPR-CAS9 and single-cell RNA-Seq informed comprehensive ex vivo evaluations of IAPi plus pan-BET, bD-selective BET, or selective BET isoform targeting in CD4^+^ T cells from ART-suppressed donors. IAPi+BETi treatment resulted in striking induction of cell-associated HIV *gag* RNA, but lesser induction of fully elongated and *tat-rev* RNA compared with T cell activation–positive controls. IAPi+BETi resulted in HIV protein induction in bulk cultures of CD4^+^ T cells using an ultrasensitive p24 assay, but did not result in enhanced viral outgrowth frequency using a standard quantitative viral outgrowth assay. This study defines HIV transcriptional elongation and splicing as important barriers to latent HIV protein expression following latency reversal, delineates the roles of BET proteins and their BDs in HIV latency, and provides a rationale for exploration of IAPi+BETi in animal models of HIV latency.

## Graphical abstract

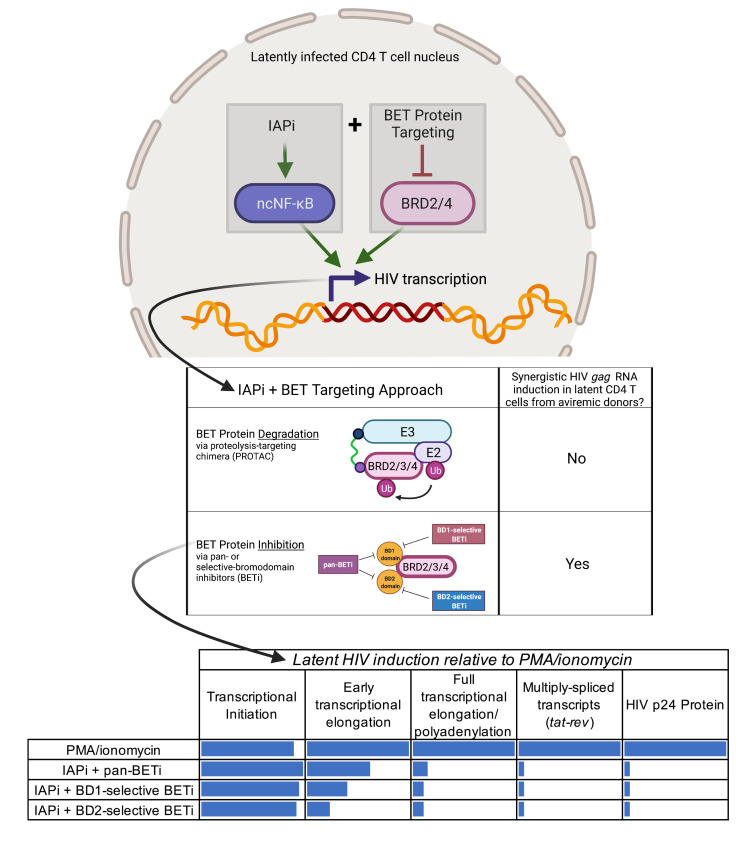

## Introduction

HIV cure remains elusive because of latent but replication-competent HIV DNA within host CD4^+^ T cells during long-term suppressive antiretroviral therapy (ART) (1, 2). HIV latency is maintained by a variety of transcriptional and epigenetic barriers as well as other barriers to viral expression (2). A prominent strategy for HIV cure is reversal of HIV latency using small molecule latency reversal agents (LRAs) that promote HIV expression paired with augmented immune clearance to eliminate latently infected cells (2).

While understanding of factors that regulate HIV latency has yielded an array of targets for LRA development, there has been limited clinical progress. This may be due to inadequate levels of latency reversal with current LRAs tested in humans to date, including histone deacetylase inhibitors (HDACi) and innate immune agonists (2). These first-generation LRAs may only reactivate a minority of the population of all proviruses in vitro, although it is currently unclear what fraction of a population is absolutely unresponsive to longitudinal LRA exposures in vivo (3). Further, whether the HIV RNA inductions observed with LRAs tested clinically to date translates to sufficient viral protein expression for an adequate period of time to engage immune effectors in vivo is unclear (2).

An emerging approach to augmenting HIV latency reversal utilizes inhibitor of apoptosis protein (IAP) antagonist (IAPi) to trigger noncanonical NF-κB signaling and drive HIV transcription. IAPi induces on-ART plasma viremia in animal models of HIV latency, which is one of the most profound latency reversal phenotypes observed in vivo to date (4, 5). However, expectations for IAPi are tempered by ex vivo studies of IAPi in CD4^+^ T cells from ART-suppressed HIV-seropositive participants, which indicate an HIV induction profile similar to that of existing LRAs, such as HDACi (4, 6, 7).

It is unclear whether activation of noncanonical NF-κB signaling will be sufficient as a single mechanism to induce proviral expression in all latently infected cells (2). IAPi promote HIV expression through induction of the noncanonical NF-κB transcription factor (8). However, in primary CD4^+^ T cells, there are several other mechanisms that maintain HIV latency, including negative regulation of active positive transcription elongation factor (P-TEFb), low levels of the viral transactivator of transcription (Tat), and a restrictive chromatin environment surrounding the HIV promoter (2, 9). Efficacious LRA regimens may need to target most if not all of these barriers in parallel or in sequence and likely at multiple points in time given the dynamic nature of chromatin in host CD4^+^ T cells and its influence on proviral expression (10–16).

Ultimately, human trials with IAPi and efficacious immune clearance agents are needed to determine whether additional LRAs are required to facilitate HIV reservoir depletion. However, given the notable discrepancy between the degree of IAPi latency reversal observed in animal models compared with ex vivo studies of human cells and the known barriers to latent HIV expression that are not targeted by IAPi, we comprehensively evaluated the efficacy of IAPi-based combination LRA regimens in cell line models of latency and primary CD4^+^ T cells from ART-suppressed donors (2, 4, 9). This study builds upon the excellent work of other groups showing latency reversal synergies with other LRA combination strategies (reviewed in ref. 17), but with the key advantage of using IAPi for stimulation of NF-κB signaling rather than PKC agonists, which have a narrow therapeutic index (18).

## Results

### Evaluation of IAPi-based combination latency reversal regimens.

**Figure 1:**
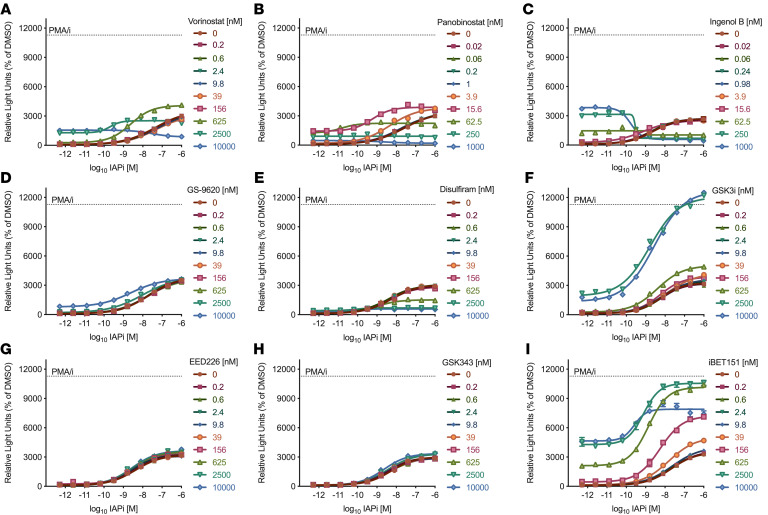
Evaluation of IAPi-based combination latency reversal regimens in the triple Jurkat model. Cross titration of IAPi with (**A** and **B**) HDACi vorinostat (**A**); panobinostat (**B**); (**C**) PKC agonist ingenol B; (**D**) TLR-7 agonist GS-9620; (**E**) disulfiram; (**F**) GSK3i; (**G** and **H**) PRC2-targeted histone methyltransferase inhibitors EED226 (**G**) and GSK343 (**H**); and (**I**) BET inhibitor I-BET151. The *y* axes represent proviral luciferase reporter induction following 48 hours of drug exposure, normalized to DMSO-treated cells on each plate. Dose-response curves were generated using GraphPad Prism 9 using a log(agonist) versus response variable slope 4 parameter curve fit. Each point represents the average signal from 2 replicate wells from a single targeted screen experiment. Different colored curves represent a fixed concentration, indicated in the figure panels, of LRA combination partner with dose titration of IAPi. Conditions without IAPi treatment were plotted as 5 × 10^–13^ M for visualization on the log_10_ *x* axis.

IAPi-based combination LRA regimens were evaluated in a triple Jurkat cell line model of latency. This model is composed of 3 independent latently infected clones containing replication-competent proviruses with luciferase reporters in place of *nef* (4). Cross titrations of the IAPi compound AZD5582 (referred to as IAPi for brevity) with LRAs of several distinct mechanistic classes were evaluated (Figure 1). Fixed concentrations of each LRA in combination with a titration of IAPi were plotted to evaluate combined activity; left and/or upward shift of the IAPi dose-response curves indicated enhanced latency reversal. Confirmatory experiments to verify combination latency reversal activity (or lack thereof) and assess toxicity (CellTiterGlo assay for total cellular ATP) were also performed (data not shown).

In this Jurkat model of latency, there was some enhanced activity for IAPi+HDACi; however, this occurred at relatively high exposures of HDACi: 625 nM vorinostat and 4 nM panobinostat showed combination activity with IAPi, but the higher doses demonstrated overt toxicity (defined as >50% decline in total cellular ATP levels; Figure 1, A and B). Combination of IAPi with the PKC agonist ingenol B resulted in antagonism of latency reversal. Ingenol B demonstrated overt toxicity even at active single-agent doses, which appeared to be exacerbated by IAPi (Figure 1C). Testing of the innate-immune TLR7 agonist GS-9620 did not result in combination activity or toxicity (Figure 1D and ref. 19). Disulfiram, an Akt-signaling pathway activator, did not demonstrate combination activity and, at the higher doses, resulted in antagonism of IAPi activity, which was associated with toxicity (Figure 1E and ref. 20). Combination latency reversal activity was observed for higher doses of a glycogen synthase kinase 3 inhibitor (GSK3i), SB-698596-AC (ref. 21 and Figure 1F). Two polycomb repressive complex 2–targeted LRAs, EED antagonist EED226 and EZH2 inhibitor GSK343, did not demonstrate obvious latency reversal or overt toxicity as single agents or in combination with IAPi (Figure 1, G and H, and refs. 22–24).

Striking combination activity was observed at a range of concentrations for 2 bromodomain (BD) and extraterminal domain protein inhibitor (BETi) compounds, JQ1 and I-BET151, in combination with IAPi (Figure 1I and data not shown). At very high exposures (≥2.5 μM for I-BET151), IAPi+BETi demonstrated overt toxicity in this cell line model. Notably, levels of proviral induction following IAPi+BETi treatment approached those observed with the HIV latency reversal positive control PKC agonist and calcium ionophore pair phorbol 12-myristate 13-acetate and ionomycin (PMA/i).

### Synergistic latency reversal activity with IAPi+BETi.

**Figure 2:**
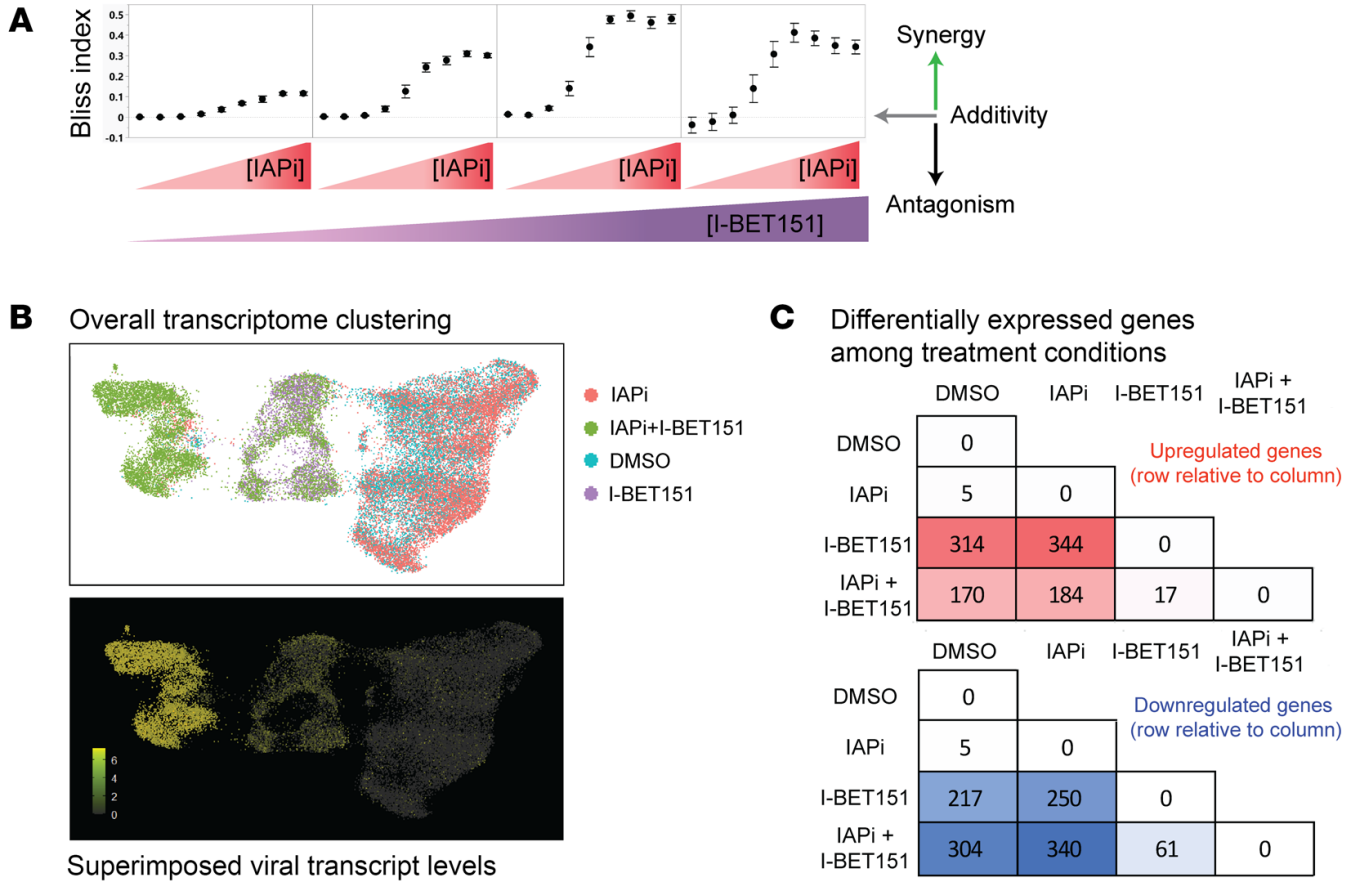
Synergistic latency reversal activity for the IAPi and BETi combination in Jurkat N6 model of latency. (**A**) Assessment of IAPi+BETi (I-BET151) latency reversal synergy using the Bliss independence model. Error bars represent SEM from pooled replicates across *n =* 2 independent experiments. (**B**) UMAP plots depicting overall transcriptome clustering and sample identity (top) and superimposed viral transcript detection (bottom) across treatment conditions. Each dot represents a single cell. Coloring of bottom panel was altered in Adobe Photoshop for ease of visualization of single cells. (**C**) Gene expression heatmap (red = upregulated; blue = downregulated) indicating the number of significantly differentially expressed genes identified in pairwise comparisons for each treatment condition. Color gradients indicate increasing numbers of statistically significant differentially expressed genes. Differential expression analysis was performed on sctransform normalized values with cutoffs for gene features expressed in at least 10% of cells and a log_2_-fold difference of at least 0.25. Statistical significance for DEGs was evaluated with genome-wide Wilcoxon’s ranked sum tests with Bonferroni’s correction.

The Bliss independence model was employed to assess synergy between IAPi and BETi (Figure 2 and ref. 25). IAPi and the BETi I-BET151 demonstrated dose-responsive increases in synergistic activity in the Jurkat model (Figure 2A). This combination was further evaluated at the single-cell level in the Jurkat N6 cell line using single-cell RNA-Seq (scRNA-Seq) to evaluate the frequency of cells undergoing reactivation and define the impact of IAPi and/or BETi on the host cell transcriptome (Figure 2, B and C).

IAPi and BETi both induce host transcriptional changes in addition to their effects on the HIV provirus (4, 26). To evaluate whether IAPi+BETi treatment also synergistically induced host transcripts, we performed scRNA-Seq of Jurkat N6 cells; this provided a high-resolution picture of host and viral gene expression. DMSO, IAPi, BETi, or IAPi+BETi conditions underwent scRNA-Seq using the 10× Genomics 3′ capture technique. 2D visualization of scRNA data was performed using uniform manifold approximation and projection (UMAP), which revealed DMSO- and IAPi-treated cells forming a distinct separate cluster as compared with I-BET151– and IAPi+I-BET151–treated cells (Figure 2B). These data suggested that I-BET151, but not IAPi, drives major transcriptome remodeling. Concordant with this, differential gene expression analysis revealed major transcriptional changes associated with BETi treatment (Figure 2C and Supplemental Data Set 1; supplemental material available online with this article; https://doi.org/10.1172/JCI157281DS1).

IAPi+BETi treatment drastically increases the number of Jurkat N6 cells undergoing latency reversal at the transcript level (97.3% with IAPi+BETi vs. 19.2% with IAPi and 12.6% with I-BET151) (Figure 2B). HIV was the most variable transcript in each treatment condition; the marked enrichment of HIV transcripts with IAPi+BETi treatment suggest the provirus is uniquely responsive to this combination relative to cellular genes. For example, CXCR4, a cellular gene that exhibits similarity to HIV provirus with respect to promoter structure, was not synergistically induced by combination treatment (Supplemental Figure 1 and ref. 27). Additionally, known IAPi-upregulated genes *BIRC3* and *NFKB2* were not further upregulated by the addition of I-BET151 in primary CD4^+^ T cells (Supplemental Figure 2 and ref. 4). Further, IAPi+BETi synergy was dependent on NF-κB sites within the HIV promoter when evaluated in 2D10 (WT NF-κB sites) and 2B5 (mutant NF-κB sites) Jurkat models of latency (Supplemental Figure 3 and ref. 28).

### BET protein KO reveals a primary role for BRD4 in IAPi+BETi combination LRA activity.

BETi target BRD2, BRD3, and BRD4 proteins with a high degree of selectivity (29). Initial reports attributed latency reversal with BETi to reduced host/viral competition for the critical HIV transcriptional coactivator P-TEFb among BRD4, the 7SK small nuclear ribonucleoprotein, and the viral protein Tat (9, 30–34). Increased access of Tat to P-TEFb facilitates super elongation complex (SEC) formation at the HIV promoter, which then drives highly processive HIV transcription (reviewed in ref. 9).

This reduced host/viral competition for P-TEFb is thought to be mediated by BETi-mediated displacement of the long isoform of BRD4 (BRD4L) from chromatin; this isoform contains a C-terminal P-TEFb–binding domain (32). Conrad and colleagues have also reported that the BETi-mediated displacement of the short isoform of BRD4 (BRD4S) may contribute to latency disruption via reduced recruitment of repressive SWI/SNF chromatin remodeling complexes to the HIV promoter (35). BRD2 inhibition may similarly reduce recruitment of repressor complexes (36). The contribution of each of these BET proteins/isoforms to latency reversal alone or with IAPi was evaluated to inform on the molecular mechanisms responsible for IAPi+BETi LRA synergy.

**Figure 3:**
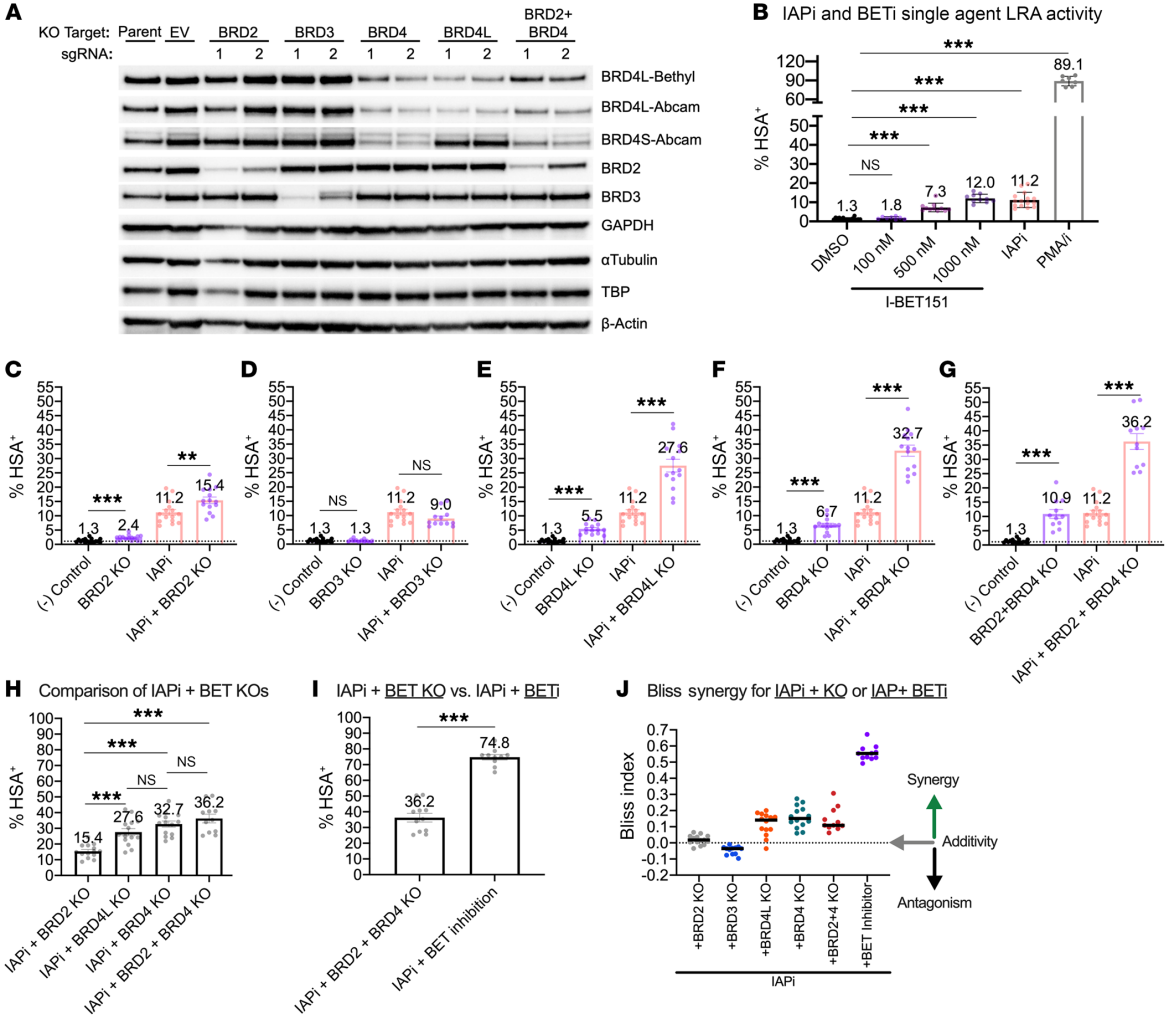
BET protein KO reveals a primary role for BRD4 in IAPi+BETi combination LRA activity in Jurkat N6 cells. (**A**) Representative Western blot following lentiviral transduction of sgRNA targeted to BET family proteins. Data are representative of 7 independent experiments, with each target quantified in at least *n =* 4 experiments (Supplemental Figure 4). Empty vector (EV) and mock-infected (parent) cells served as negative control conditions (corresponding to DMSO or (-) control labels in **B**–**G**). (**B**) HSA reporter expression on live N6 cells following 48 hours IAPi (100 nM AZD5582) or BETi (1 μM I-BET151) exposure. Error bars represent SEM. (**C**–**G**) HSA reporter expression on live single cells following 48 hours of IAPi (100 nM AZD5582) and/or BETi (1 μM I-BET151) drug treatment in the presence or absence of different BET protein or BET protein isoform KOs. (**H**) Comparison of HSA reporter expression across IAPi+BET protein KO conditions. (**I**) Comparison of IAPi+BET protein KO and inhibition. For **B**–**I**, each dot represents an HSA determination for a sgRNA targeted to the indicated protein(s) or isoform(s). Each protein or isoform was targeted with 2 sgRNAs across *n =* 6 or *n =* 7 independent experiments, depending on the target. Dots for drug-treated conditions (i.e., DMSO for negative controls or IAPi±BETi) represent HSA determinations following treatment of parent N6 and empty vector transduced cells. Error bars represent SEM. FDR-adjusted *P* values for pairwise comparisons using Wilcoxon’s rank-sum tests are indicated as follows: **P < 0.01; ***P < 0.001. (**J**) Bliss independence analysis of IAPi+BET KO conditions versus IAPi+BETi. Black bars indicate median Bliss index.

Latently infected N6 cells were transduced with CRISPR-CAS9–expressing lentiviruses with single guide RNAs (sgRNAs) directed against the BET family proteins BRD2, BRD3, BRD4L, BRD4 (both isoforms), and BRD2+BRD4 (both isoforms) to generate short-term polyclonal KOs (Figure 3A and ref. 4). Immunoblot analysis revealed similar KO levels for each target at the time of DMSO or IAPi stimulation: mean depletion, 68% (BRD2), 85% (BRD3), 70% (BRD4), 72% (BRD4L), and 69% (averaged across BRD2+BRD4) (Supplemental Figure 4). To provide context for the latency reversal effect of BET protein KO, single-agent IAPi and BETi treatments were conducted in parallel (Figure 3B). Depletion of BRD2, BRD4L, BRD4 (both isoforms), BRD2+BRD4 (both isoforms), but not BRD3, caused a consistent increase in viral gene expression detected by flow cytometry for the viral HSA reporter relative to baseline HSA expression in control conditions (Figure 3, C–G).

We next assessed combination latency reversal activity by treating BRD-KO cells with IAPi (Figure 3, C–G). IAPi plus BRD2, BRD4L, BRD4 (both isoforms), BRD2+BRD4 (both isoforms), but not BRD3, KO resulted in enhanced latency reversal activity over IAPi alone (Figure 3, C–G). IAPi+BRD4L KO, IAPi+BRD4 KO, and IAPi+BRD2+4 KO resulted in greater latency reversal than IAPi+BRD2 KO (Figure 3H). IAPi+BRD4 KO resulted in numerically larger proviral induction than IAPi+BRD4L KO, but was not statistically different. The same trend occurred for the comparison of IAPi+BRD2+4 KO with IAPi+BRD4 KO (Figure 3H).

Interestingly, when IAPi+BRD2+4 KO was compared with IAPi+BETi, the use of BETi resulted in greater latency reversal (Figure 3I). Nevertheless, these results are consistent with previous literature observing distinct contributions to latency maintenance of the BRD2 protein and the BRD4S and BRD4L isoforms (32, 35, 36). Bliss independence analysis of the IAPi+BETi KO studies demonstrated a greater synergy index for conditions that target BRD4 compared with BRD2 or BRD3 (Figure 3J). Therefore, at least in this cell line system, IAPi+BETi combination activity relies mainly on BRD4 targeting.

### Selective targeting of BRD4 alone or in combination with IAPi in primary CD4^+^ T cells from aviremic donors.

**Figure 4:**
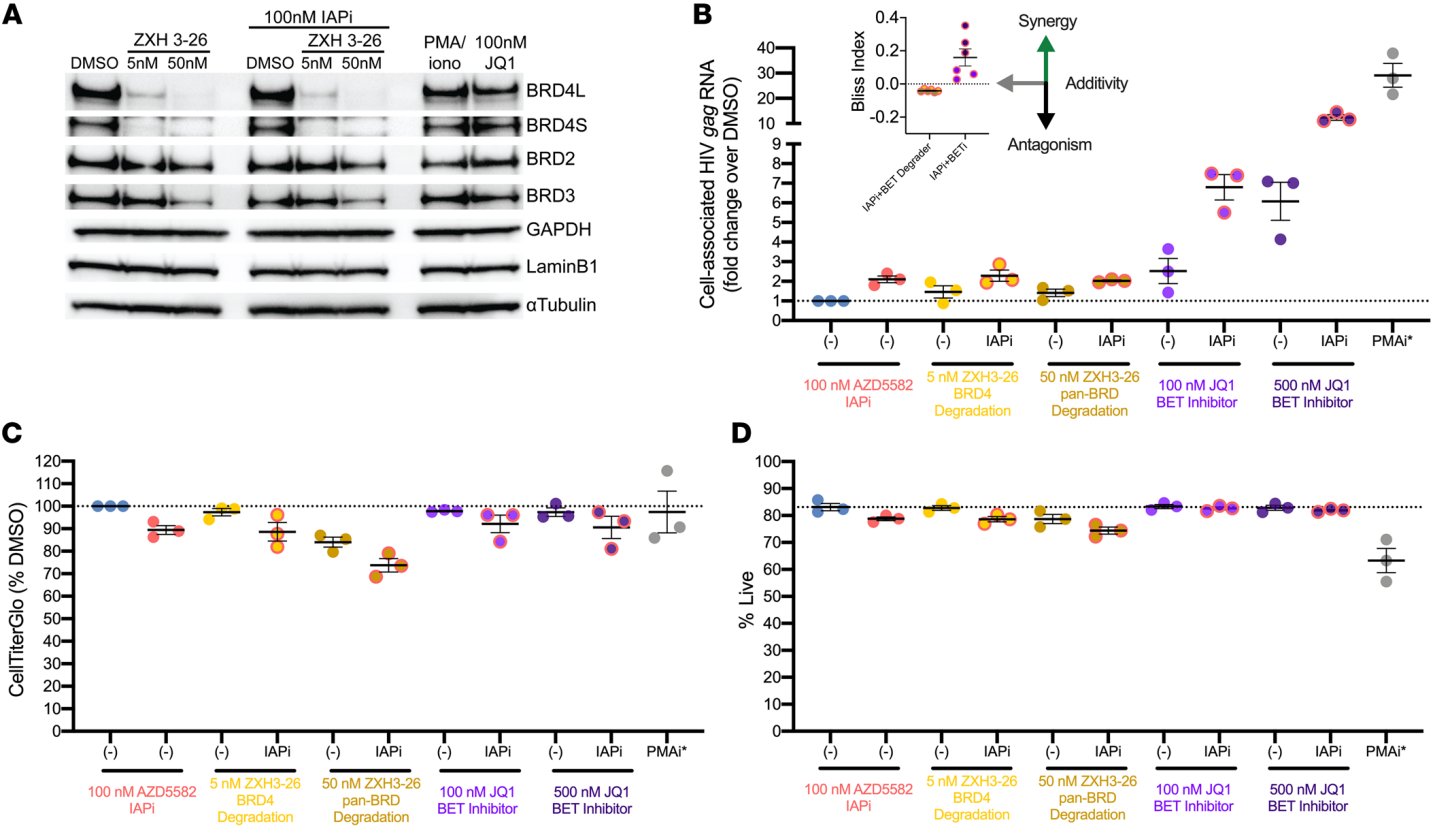
Selective targeting of BRD4 alone or in combination with IAPi in primary CD4^+^ T cells from aviremic donors. (**A**) Western blot of BET family proteins showing BRD4-specific degradation with 5 nM of the BRD PROTAC ZXH 3-26. BET degradation was confirmed by Western blot for all (*n =* 3) donors; a representative blot is shown. (**B**) Fold change in HIV *gag* cell–associated RNA (normalized to *TBP* expression, except for PMA/i due to known *TBP* upregulation following PMA/i exposure; ref. 78) in total CD4^+^ T cells following 24 hours of exposure to the indicated compounds. Each dot represents the average induction of 4 to 5 replicates of 1 to 2 million CD4^+^ T cells for an individual donor (*n =* 3). Error bars represent SEM. Bliss index for combination regimens is indicated in the upper left. (**C**) Total cellular ATP levels normalized to DMSO and (**D**) percentage of live cells measured by AOPI membrane exclusion dye microscopy-based assay following drug exposure. Note that PMA/i viability with AO/PI staining may be an underestimate of viability due to the formation of large clusters of proliferating viable cells. Each dot represents the average of 4 to 5 replicates (**C**, CellTiterGlo) or 2 replicates (**D**, AO/PI staining) for each donor (*n =* 3). Error bars represent SEM. Donors A-2, B, and C were tested (Supplemental Table 1).

Next, we performed a series of evaluations of IAPi and BET-targeted LRA combinations to assess latency reversal efficacy in CD4^+^ T cells from aviremic donors. Given the prominent role of BRD4 in IAPi+BETi synergy in the Jurkat model (Figure 3), we employed a BRD-selective proteolysis targeting chimera (PROTAC), ZXH3-26, that selectively degrades BRD4 at 5 nM and results in BRD2, BRD3, and BRD4 degradation at 50 nM in primary CD4^+^ T cells (Figure 4A and ref. 37).

Interestingly, despite efficient degradation of target proteins, the ZXH3-26 BET PROTAC resulted in a lesser degree of HIV *gag* cell–associated RNA induction than the nondegrading parent BETi compound JQ1 both alone and in combination with IAPi (Figure 4B). A similar phenotype of lesser latency reversal using BET PROTACs compared with inhibitors was observed in the triple Jurkat model (Supplemental Figure 5). Further, BET degraders, when used at pan-BET degrading concentrations, appeared to have a slightly greater toxicity profile than BET inhibitors using assays for total cellular ATP levels and membrane exclusion dyes (Figure 4, C and D). Taking these data together, we concluded that BET inhibition, but not degradation, synergized with IAPi; hence, we next evaluated pan- and BD-selective BET inhibitors.

### IAPi and BETi combination latency reversal activity in primary CD4^+^ T cells from aviremic donors.

BET family proteins contain 2 bromodomains (BD1 and BD2) that bind to chromatin and transcription factors (38–40). BETi tested as LRAs to date bind to both BD1 and BD2 bromodomains (pan-BETi) and displace BET proteins from chromatin. Recently, tool molecules have become available that bind to either the BD1 or BD2 bromodomains of the BRD2/3/4 proteins with a high degree of selectivity (41, 42). BD-selective inhibitors may confer a safety advantage through mitigation of the global impact of pan-BET inhibition on normal host cell processes (refs. 41, 43, and Supplemental Figure 6).

**Figure 5:**
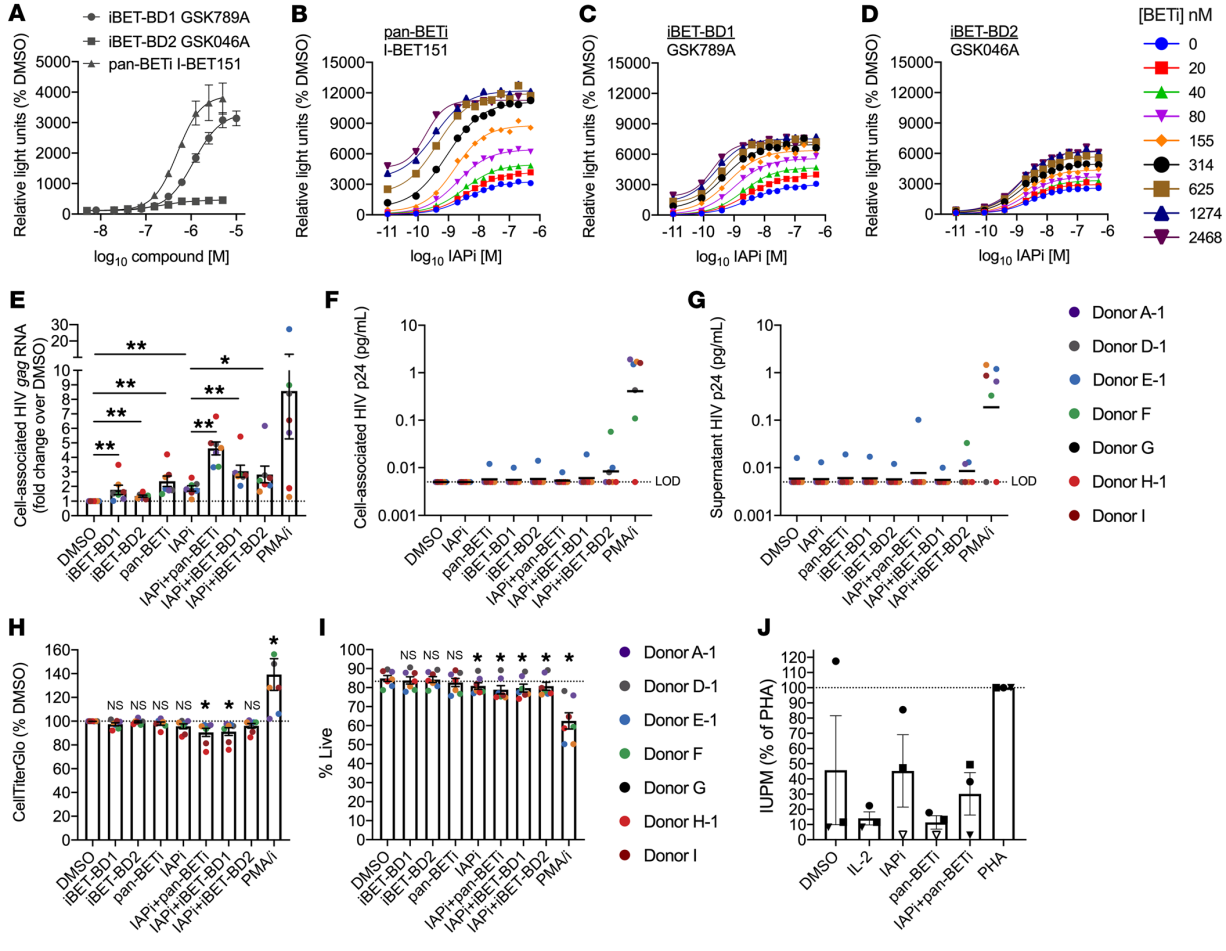
Pan or selective targeting of BET protein BD domains alone or in combination with IAPi. (**A**) Dose-response curves for pan-BD and BD-selective BETi in the triple Jurkat model across *n =* 3 independent experiments. (**B**–**D**) Combination activity of IAPi and (**B**) pan-BETi, (**C**) iBET-BD1, or (**D**) iBET-BD2 in the triple Jurkat model. Representative of *n =* 4 independent experiments. Conditions without IAPi treatment were plotted as (**A**) 5 × 10^–9^ M or (**B**–**D**) 1 × 10^–11^ M for visualization on the log_10_ *x* axis. (**E**) Resting CD4^+^ T cell HIV *gag* caRNA (*TBP* normalized, except for PMA/i due to known *TBP* upregulation following PMA/i exposure, ref. 78, **I**) with parallel measurements of cell-associated p24 protein (**F**), and (**G**) culture medium p24 protein induction following 40 hours exposure of IAPi (100 nM AZD5582), pan-BETi (1 μM I-BET151), BD1- or BD2-selective BETi (2 μM), or combinations thereof compared with the positive control PMA/i. Horizontal black lines indicate (**E**) mean ± SEM or (**F** and **G**) geometric mean across all donors. (**H**) Total cellular ATP levels and (**I**) cellular viability following 40 hours drug exposure. Note that PMA/i viability with AO/PI staining may be an underestimate of viability due to the formation of large clusters of proliferating viable cells. (**J**) QVOA following IAPi and pan-BETi exposure relative to the positive control PHA/IL-2. Infectious unit per million resting CD4^+^ T cells (IUPM) for each condition represented as a percentage of the PHA IUPM for each donor (different shapes). Open shapes indicate no positive wells were detected. For QVOA, resting CD4^+^ T cells from donors E-2, G, and D-2 were evaluated (Supplemental Table 1). All error bars represent mean ± SEM. FDR-corrected *P* values for pairwise comparisons using Wilcoxon’s signed-rank tests are indicated as follows: *P < 0.05; **P < 0.01.

Pan-BETi (I-BET151) and BD-selective BETi (iBET-BD1; GSK789) and iBET-BD2 (GSK046) were evaluated in the triple Jurkat model of latency (Figure 5A and ref. 4, 41, 42). iBET-BD1 demonstrated greater latency reversal activity than iBET-BD2 (Figure 5A). When tested with IAPi, there was combination latency reversal activity with both selective inhibitors (iBET-BD1 > iBET-BD2), albeit to a lesser extent than with pan-BETi (Figure 5, B–D).

Next, IAPi and pan- and selective BETi were evaluated in primary resting CD4^+^ T cells from ART-suppressed donors. Concentrations of I-BET151, iBET-BD1, and iBET-BD2 were chosen to target approximately 85% inhibition of the respective BD(s) based on time-resolved fluorescence energy transfer and other assays (data not shown). Given the relatively slow kinetics of noncanonical NF-κB signaling, we first used a 40-hour drug exposure and assessed HIV cell–associated *gag* RNA induction and p24 protein induction (4, 8).

High levels of HIV *gag* RNA induction were observed for the latency reversal positive control PMA/i with the exception of 2 donors, possibly due to toxicity associated with prolonged PMA/i exposure (Figure 5E). Single-agent IAPi and pan-BETi conditions resulted in approximately 2-fold inductions of HIV cell–associated *gag* RNA, with lesser but still significant LRA activity for iBET-BD1 and iBET-BD2 (Figure 5E). Combination IAPi+BETi treatment enhanced latency reversal as compared with IAPi single-agent treatment, with the largest effect observed for IAPi+pan-BETi, which resulted in a median 4.7-fold induction of HIV cell–associated *gag* RNA (Figure 5E).

Based on the remarkable up to 14-fold induction (depending on donor, BETi compound, and time point assayed; Figure 4B and Figure 5E) of cell-associated HIV *gag* RNA following IAPi+pan-BETi exposure, we anticipated that HIV protein expression was likely also induced. Cell-associated and supernatant HIV p24 levels were measured in parallel using an ultrasensitive combined immunoprecipitation and digital ELISA methodology (Figure 5, F and G, and ref. 44). As reported previously, there was some spontaneous release of p24 in a minority of donors (44). Although high levels of p24 were detected reliably with PMA/i, there was not consistent strong induction of HIV p24 following IAPi and pan- or BD-selective BETi exposure in primary resting CD4^+^ T cells. IAPi+BD2-BETi resulted in p24 induction in a few donors, but the biological significance of this low-level induction is unclear, given the proximity to the limit of detection of the assay. Importantly, the latency reversal observed with IAPi+BETi occurred with marginal (median difference < 5% compared with DMSO) impact on cellular viability or total cellular ATP despite a relatively long drug exposure (Figure 5, H and I).

Despite the low levels of HIV protein induction, we sought to independently determine whether IAPi+pan-BETi induces viral outgrowth ex vivo. We performed quantitative viral outgrowth assays (QVOA) using IAPi+pan-BETi and single agents in resting CD4^+^ T cells from 3 aviremic donors (Figure 5J). Positive control phytohaemagglutinin (PHA)+IL-2 mitogen stimulation induced robust viral outgrowth. However, although there was fractional activation of the reservoir with IAPi alone, as observed previously in some donors (4), there was not clear or consistently enhanced frequency of outgrowth with IAPi+I-BET151 combination treatment (Figure 5J).

### Mechanistic evaluations of the HIV gag RNA-protein disconnect.

The finding of strong cell-associated HIV unspliced *gag* RNA induction, yet relatively low p24 protein induction even with ultrasensitive detection methods, prompted exploration of this apparent disconnect between viral RNA and protein levels. One possibility is that IAPi and/or BETi impairs the host translation machinery. Using an assay for nascent translation, we found that, while BETi slightly reduced rates of nascent translation in primary CD4^+^ T cells, there was not a significant global impairment with IAPi and/or BETi, as was observed with the translational elongation inhibitor cycloheximide (Supplemental Figure 7). Notably, we observed markedly increased levels of translation in PMA/i-stimulated compared with unstimulated primary CD4^+^ T cells.

Another potential explanation for the lack of HIV protein induction is inefficient nuclear export of HIV RNA (45). However, nuclear and cytoplasmic RNA fractionations of primary CD4^+^ T cells from ART-suppressed donors demonstrated upregulation of HIV *gag* RNA in both nuclear and cytoplasmic fractions following IAPi+BETi treatment (Supplemental Figure 8). Fractionation efficiency was confirmed using unspliced/spliced *GAPDH* and the 40S ribosomal protein S14 (*RPS14*) RNA transcripts as controls as well as Western blot confirmation of efficient cellular fractionation at the protein level prior to RNA extraction (Supplemental Figure 8).

Finally, the discrepancy between strong induction of *gag* cell–associated HIV RNA, but lesser p24 protein induction, might be explained by a failure of IAPi+BETi treatment to overcome barriers to HIV transcriptional elongation, completion, and splicing. To evaluate this possibility, an HIV transcript profiling assay was conducted to absolutely quantify induction of 7 classes of HIV transcripts (TAR, read through, elongated LTR, *pol*, *tat-rev*, polyadenylated, and fully elongated [*nef*]; ref. 46). First described by Yukl and colleagues, this assay employs carefully optimized cDNA synthesis and digital PCR to enable reasonable comparisons of transcript induction magnitude across different regions of the HIV genome (46). Having established proof-of-concept LRA combination activity with long drug exposures (24–40 hours; Figures 4 and 5), these more detailed transcript profiling experiments were performed with an 8-hour drug treatment to more accurately recapitulate an in vivo BETi exposure (29, 41).

Across 5 donors, DMSO-treated aviremic donor CD4^+^ T cells had high frequencies of 5′ transcripts (TAR, elongated LTR, and *pol*) but 1 to 2 log_10_ lower levels of multiply spliced, polyadenylated, and fully elongated transcripts, similar to what was originally reported with this assay (Supplemental Figure 9 and ref. 46). The highest levels of induction for IAPi and/or BETi were observed for elongated LTR transcripts (median 6.5-fold induction with IAP+I-BET151, 4.7-fold induction with IAPi+iBET-BD1, and 2.7-fold induction with IAPi+iBET-BD2). Relative to elongated LTR and *pol* transcripts, there was lesser induction of fully elongated polyadenylated and *nef* transcripts (median approximately 3.5-fold induction with IAP/I-BET151, and median approximately 2-fold inductions with the IAPi+BD-selective BETi for both polyadenylated and *nef* transcripts) (Figure 6A). Given lesser fully elongated transcript induction with IAPi+BETi, we asked whether IAPi+BETi impair HIV transcriptional elongation in the Jurkat N6 cell line, which, unlike cells from aviremic patients, contains a single intact provirus in each cell. Assessment of transcripts (*gag*, *env*, post-A7, U3) in the scRNA-Seq data following IAPi+BETi did not demonstrate clear decreases in the percentages of fully elongated transcripts (i.e., U3) compared with less elongated transcripts (i.e., *gag*) as a percentage of total HIV reads for DMSO versus IAPi+BETi treatment (Supplemental Figure 10).

Completion of transcriptional elongation and the generation of multiply spliced *tat-rev* transcripts are essential requirements of HIV latency reversal, as these transcripts are required for robust initiation of a viral positive transcriptional feedback loop (9, 47). Depending on the primary cell donor, there was minimal to no induction (median 1.5-fold) of multiply spliced *tat-rev* transcripts with IAPi+I-BET151, with similar findings for the IAPi+BD-selective BETi (Figure 6A). Extremely low levels of *tat*-*rev* transcripts preclude strong conclusions about the presence or absence of small inductions following IAPi+BETi exposure in some donors due to greater levels of assay noise at low absolute levels of transcript. However, PMA/i treatment consistently resulted in indisputable *tat-rev* inductions. Additionally, Bliss independence analysis of this data set revealed synergistic activity of IAPi+pan-BETi on TAR, elongated LTR, and *pol* transcripts, while additive effects were observed on polyA, *nef*, and *tat-rev* transcripts (Figure 6B). Similar findings were observed for IAPi+iBET-BD1, while IAPi+iBET-BD2 demonstrated synergy only when TAR transcripts were measured, with additive effects for the other transcripts. Taken together, these data suggest that while IAPi+BETi shows strong synergy on HIV transcript initiation and early elongation, the IAPi+BETi combination is less efficient at inducing full transcriptional elongation, polyadenylation, and splicing of HIV transcripts.

To contextualize these transcript findings (Figure 6, A and B) with HIV protein induction levels, approximately 5 million CD4^+^ T cells per condition (such that there were many replication-competent inducible reservoir cells in each drug treatment condition based on previously determined QVOAs) were extensively washed following 8 hours of drug exposure and cultured with serial sampling of the culture supernatant for HIV p24 protein using an ultrasensitive digital ELISA method (Figure 6C and ref. 7). PMA/i resulted in consistent p24 induction across all 4 donors tested. As observed above in longer exposure periods (Figure 5, E–G), IAPi+BETi treatment resulted in inconsistent p24 detection across donors and at levels 1 to 2 log_10_ lower than those observed for PMA/i-treated cells (Figure 6C). In general, p24 protein was more likely to be detected in combination treatments, but with the relatively sporadic induction of p24, no clear pattern emerged as to whether IAPi+pan-BETi, IAPi+iBET-BD1, or IAPi+iBET-BD2 exhibited superior p24 protein induction.

Taken together, these data suggest that, while the IAPi+BETi LRA combination strongly induces HIV transcripts containing *gag,* lesser generation of fully elongated/polyadenylated/multiply spliced transcripts occurs and is associated with limited HIV p24 protein induction. In contrast, PMA/i treatment substantially increases all HIV transcript species and is associated with high levels of p24 protein induction.

To further confirm this finding of decreased full HIV transcriptional elongation and polyadenylation in primary cells (Figure 6), we performed an orthogonal assay to measure the induction of polyadenylated versus total viral RNA transcripts. Magnetic beads coated with polydT oligos were employed to generate a polyA-captured RNA fraction from the CD4^+^ T cells of ART-suppressed donors following an 8-hour IAPi+BETi or DMSO treatment. Consistent with the transcript profiling assay results (Figure 6), there was decreased *gag* induction in the polyA RNA fraction relative to the total RNA fraction (Supplemental Figure 11). As a comparison, we examined polyadenylation of the *NFKB2* gene, a defined transcriptional target of IAPi, after IAPi+BETi treatment in the same fractions (4). Polyadenylation of *NFKB2* transcripts appeared unperturbed, suggesting that the decreased elongation and polyadenylation of transcripts following IAPi+BETi treatment may be HIV specific.

## Discussion

Recent preclinical work has demonstrated that IAPis induce unprecedented levels of plasma viremia in HIV-infected humanized mouse and SIV-infected macaque models in the presence of suppressive ART (4). However, it is still likely that combination approaches will be needed to fully target the latent HIV reservoir.

### Potential combination LRA partners for IAPi.

There were several notable findings from the initial combination LRA screen in the Jurkat model of latency (Figure 1). IAPi showed combination activity with HDACi at higher drug exposures. This combination could be further evaluated in primary cells for combination LRA activity with drug exposures that might be achievable in vivo (8). IAPi did not demonstrate combination activity with the PKC agonist ingenol B at least in part due to toxicity. Evaluation of IAPi with the innate immune agonist GS-9620 did not demonstrate combination activity in this Jurkat model. Evaluation of other TLR agonists that directly target T cells might be warranted (48). Latency reversal was not observed for the PRC2-targeted histone methyltransferase inhibitors EED226 or GSK343 alone or in combination with IAPi at drug exposures tested in this Jurkat model. Previous data suggest these inhibitors demonstrate LRA activity both in vitro and ex vivo using more prolonged (3 to 4 day) exposures (22–24). The contributions of histone methyltransferases to HIV latency may also vary depending on the cell line model utilized (22–24). At higher doses, there was combination activity between IAPi and the GSK3i SB-698596-AC (21). GSK3i have single-agent latency reversal activity in aviremic donor cells and might be further evaluated with IAPi (49).

### LRA efficacy of IAPi, BETi, and BET degraders.

The most profound latency reversal phenotype observed in the targeted screen was the combination of IAPi+BETi (Figure 2). Cell-line models of HIV latency do not completely recapitulate latency that is observed in vivo in ART-suppressed human donors; therefore, the activity of the IAPi+BETi combination ex vivo in CD4^+^ T cells from aviremic donors was evaluated (50). Based on the observation that BRD4 targeting was responsible for most of the IAPi+BETi combination activity in the Jurkat cell-line model, we first utilized a carefully titrated BET PROTAC, ZXH3-26, to degrade either BRD4 or all BET family proteins in primary CD4^+^ T cells (37). Compared with BET inhibitors, the PROTAC BET degrader demonstrated weaker LRA activity with or without IAPi at BRD4-selective and nonselective doses (Figure 4). This is also consistent with greater activity of IAPi+BETi compared with IAPi+BET protein KOs in the Jurkat model (Figure 3).

One explanation for reduced LRA activity with BET degraders compared with inhibitors is that BET inhibitors reverse HIV latency via off-target binding to other proteins; however, extensive chemoproteomic and other methods for assaying target specificity of the BETi compounds suggest they are highly specific for BET family proteins, making this explanation less likely (29, 41). More likely, this finding suggests that a BD-domain independent function of BET proteins contributes to latency reversal. This explanation is also supported by recent work demonstrating that BET degraders, compared with BETi, result in much greater impairments in transcriptional elongation complex assembly and RNA processing (51, 52). Incomplete target degradation may also contribute to the reduced latency reversal signal observed with IAPi+BET-degrading reagents compared with BETi.

BETi appear to have greater latency reversal efficacy compared with BET degraders; however, pan-BETis that inhibit both BD domains (BD1 and BD2) fundamentally alter host cell transcription programming (Figure 2). Further, pan-BETis tested primarily in the oncology space to date have dose-limiting toxicities, including severe cytopenias that may not be acceptable for otherwise healthy people with HIV, and reproductive toxicity of BRDT inhibition is also of concern (53, 54). Preclinical data indicate that the use of BD2-selective BETi may at least partially mitigate some toxicities (43). Thus, use of BETi that target a single BD may confer a safety advantage (41, 43, 55).

To this end, LRA activity of pan-, BD1-, and BD2-selective BETis were evaluated alone in combination with IAPi (Figure 5 and Figure 6). IAPi+BETi induce large increases in cell-associated elongated LTR transcripts, with synergistic increases seen with IAPi+pan-BETi or iBET-BD1 and additive increases with IAPi+iBET-BD2 (Figure 6). Greater latency reversal activity with IAPi+iBET-BD1 (Figure 5 and Figure 6), which more efficiently displaces BRD4 from chromatin compared with iBET-BD2 (Supplemental Figure 6), is consistent with the model that displacement of BRD4S and BRD4L from chromatin drives BETi-mediated latency reversal (35, 41). When *tat-rev*, polyA*,* and *nef* transcripts were examined, smaller, additive inductions were observed with less clear differences among the IAPi+pan-BET, IAPi+iBET-BD1, and IAPI+iBET-BD2 conditions.

In contrast to what was observed in the cell-line model (Figure 1, Figure 2, and Figure 3), these large increases in HIV *gag* RNA did not consistently translate to large increases in HIV protein in aviremic donor primary CD4^+^ T cells. That said, in general, HIV p24 protein was more likely to be detected in the combination treatments relative to single agents, albeit at much lower levels than with PMA/i. No clear pattern emerged as to whether IAPi plus pan-BETi, iBET-BD1, or iBET-BD2 resulted in more HIV p24 protein production across the donors tested, but this deserves further study.

### Evaluation of potential barriers to HIV protein induction following latency reversal: translation of RNA.

To evaluate whether IAPi and/or BETi impair host translational machinery, global levels of nascent translation of cellular RNAs were measured in primary CD4^+^ T cells. No striking impairment of nascent translation with IAPi and/or various BETi treatment was observed. However, PMA/i treatment, which demonstrates robust p24 protein induction, also showed marked increases in translation. This correlative finding points to the need to further explore the role of host translational machinery in the paucity of HIV p24 production despite increased *gag* RNA transcription. The role of cellular microRNAs in preventing HIV RNA translation following IAPi+BETi exposure might also be evaluated (56).

### Nuclear export of RNA.

One report suggests that both multiply spliced and unspliced HIV RNAs are retained in the nucleus of primary CD4^+^ T cells (45). However, in this study, HIV unspliced *gag* RNA was readily detected in both nuclear and cytoplasmic fractions and increased upon IAPi+BETi treatment (Supplemental Figure 8). Robust cellular fractionation controls in these experiments suggest that it is unlikely this finding is attributable to nuclear contamination of cytoplasmic fractions.

Low levels of Rev proteins in latently infected cells that transport unspliced HIV RNAs out of the nucleus may explain this finding (57). Another possible contributor is proviruses with deletions and/or mutations in cis-acting repressive sequences, which normally function to facilitate nuclear retention of unspliced HIV RNAs (58, 59). An alternative explanation for this phenomenon is Rev-independent export of unspliced HIV *gag* transcripts. We note that this is not the first study to report detection of unspliced HIV transcripts in the cytoplasm of cell line or primary lymphocytes despite low levels of or the absence of functional Rev (60–62). Additionally, recent evidence suggests the possibility of orchestrated intron retention and cytoplasmic nonsense–mediated decay as a mechanism of gene expression control in immune cells (63). Further study of nuclear export of different HIV transcripts in aviremic donor CD4^+^ T cells is required to differentiate among these possibilities (64).

### HIV transcriptional elongation and polyadenylation.

Detection of HIV *gag* RNA indicates that RNA polymerase II has proceeded past its proximal pause point; however, HIV transcript profiling of IAPi+BETi–treated aviremic donor CD4^+^ T cells demonstrated lesser induction of fully elongated RNA and polyadenylated RNAs relative to *gag* (i.e., approximately 7-fold induction following IAPi+pan-BETi for long LTR transcripts compared with approximately 3 fold induction for polyA and *nef* transcripts) (Figure 6). In mammalian genes, release of elongating RNA pol II from its proximal pause to maximal productive elongation is not a binary on/off event, but rather a dynamic process that varies in efficiency across the gene body due to effects of nucleotide content, nucleosomes/histone marks, and splicing/termination factors (65, 66). Elongation efficiency across different proviruses could vary depending on histone marks associated with the proviral integration site, proviral diversity within polyA signal/regulatory sequences, and mutations in splice sites, among other factors (16, 46, 65, 66). These factors may decrease the efficiency of elongation/polyadenylation or potentially even result in premature transcriptional termination within the gene body (46, 65, 66).

### HIV splicing.

HIV splicing is a relatively inefficient process that results in a high ratio of unspliced to multiply spliced HIV RNA in ART-treated individuals (67–69). Compared with PMA/i stimulation, there was marginal multiply spliced *tat-rev* induction following IAPi+BETi treatment, suggestive of a reversible barrier to HIV splicing that is not efficiently overcome by IAPi+BETi (46). This barrier to HIV splicing could involve host factors such as spliceosomes and other transcriptional regulators (70). Additionally, proviral sequence features, such as inefficient splice acceptor sites, likely contribute to low levels of multiply spliced HIV RNA production (46, 68). The interactome of different HIV RNAs may also contribute to splicing, stability, export, and translation differences (64).

An alternative explanation for the paucity of *tat-rev* induction with IAPi+BETi is that BETi impair splicing of HIV RNAs. BETi are known to alter splicing patterns; however, the finding that BETis augment PKC-induced viral release from latently infected cells suggests that HIV splicing may not be markedly impaired with BETi (71, 72). It is also possible that IAPi+BETi drives expression of defective proviruses that are unable to produce *tat-rev* transcripts, although it is not readily apparent why IAPi+BETi would have a bias for induction of defective proviruses compared with PMA/i, which induces high levels of *tat-rev* transcripts within aviremic CD4^+^ T cells from the same donors.

Despite low *tat-rev* transcript and p24 protein induction at the 8-hour time point in these experiments, serial sampling of culture supernatant following LRA exposure demonstrated low-level viral protein detection with IAPi+BETi in some donors. Inefficient *tat-rev* transcript induction sufficient for protein expression in only a fraction of p24 protein-inducible reservoir cells within the culture could explain this inconsistent and low-level p24 detection (relative to PMA/i) observed following IAPi+BETi in bulk cultures of CD4^+^ T cells as well as the lack of increased frequency of viral outgrowth following IAPi+BETi treatment. The absence of robust *tat-rev* induction with IAPi+BETi in aviremic donor CD4^+^ T cells may also at least partially explain the discrepancy between robust viral reporter protein induction in Jurkat cells and relatively modest HIV p24 protein induction in aviremic donor cells (67).

### Future directions.

Consideration of the potential mechanisms of IAPi+BETi-mediated HIV transcription in aviremic donor CD4^+^ T cells in the absence of high levels of Tat may provide useful insight into development of future approaches to augmenting Tat induction (9, 47). BETi are thought to increase viral gene expression through BRD4L inhibition (via Tat-dependent transcriptional elongation through increased availability of P-TEFb to Tat), but also through BRD4S and/or BRD2 inhibition (via displacement of repressive chromatin remodeling complexes; refs. 30–36).

Following IAPi+BETi coadministration, the aviremic donor CD4^+^ T cell HIV transcript profiling data are consistent with higher levels of transcriptional initiation and initial elongation, but limited levels of Tat-dependent full transcriptional elongation (Figure 6). Therefore, we speculate that there is a significant initial role for the BETi-mediated displacement of BRD4S and/or BRD2 and the associated repressive chromatin remodeling complexes in the IAPi+BETi-mediated synergistic induction of initiated TAR and elongated LTR transcripts in aviremic donor cells (35, 36). Subsequently, in latent cells where sufficient levels of Tat are achieved following this initial IAPi+BETi transcriptional stimulus, the BETi-mediated displacement of BRD4L enables increased access of Tat to P-TEFb, followed by strong Tat-driven HIV transcription and viral protein production (47).

However, low *tat-rev* transcript induction, minimal p24 production, and lack of enhanced viral outgrowth in resting CD4^+^ T cells suggest the IAPi+BETi combination is inefficient at initiating the Tat-transcriptional positive feedback loop relative to PMA/i, although it still does so better than IAPi or BETi alone. This inefficiency could be due to restricted levels of P-TEFb in primary aviremic donor CD4^+^ T cells, among other reasons (9, 47, 73). Stochastic effects on full HIV transcriptional elongation and splicing in the absence of Tat could also play a role, driven by variations in integration site, chromatin state, transcription factor density, and other factors across different proviruses (9, 16, 73–75).

Therefore, we predict that pairing IAPi+BETi with methods to increase P-TEFb biogenesis would markedly improve HIV p24 protein production across a broader population of latent cells (73). Similarly, repeated IAPi+BETi exposures over time might overcome stochastic variations in HIV transcriptional elongation and splicing and increase the likelihood of achieving sufficient *tat* production and subsequent protein production within a single latently infected cell. Novel techniques to interrogate the transcriptional activity of single proviruses in the context of their DNA sequence, integration site, and chromatin environment will further inform on the various epigenetic and transcriptional mechanisms that could be targeted to achieving Tat induction and full proviral expression (9, 16). In addition, future work characterizing the noncanonical NF-κB transcriptional machinery is underway in order to identify therapeutic targets to rationally design LRA combination partners for IAPi, with a focus on achieving full transcriptional elongation and splicing of expressed proviruses.

### Conclusions.

IAPi+BETi, but not BET degraders, consistently induced high levels of HIV *gag* RNA expression in ART-suppressed donor CD4^+^ T cells ex vivo. However, relative to *gag*, lesser inductions of fully elongated/multiply spliced HIV RNA and p24 protein occurred. That said, p24 protein induction was observed more frequently with IAPi+BETi combinations than with single-agent treatments ex vivo in bulk cultures of CD4^+^ T cells using ultrasensitive assays. However, this was not associated with enhanced frequency of viral outgrowth using a standard QVOA.

Recent data from animal models of HIV/SIV latency highlight the possibility that ex vivo studies of human cells may not fully predict LRA activity in vivo. For example, IAPi induce relatively limited HIV RNA and p24 protein in human cells ex vivo, but profound plasma viremia in animal models of HIV latency (4, 6). Therefore, the ex vivo IAPi+BETi combination activity and additional potential in vivo LRA effects of IAPi and/or BETi provide a rationale for exploration of IAPi+BETi in animal models of HIV latency.

Conversely, animal models may not always predict the activity of LRAs in humans, given differences in the length of ART suppression and other viral reservoir/immune parameters (76). Whether IAPi will induce plasma viremia and/or sufficient latency reversal to enable immune clearance of infected cells in human participants with HIV remains to be determined in clinical studies. The studies herein highlight the importance of evaluating LRA activity in ex vivo studies of human cells to inform on LRA activity on cells containing the true (nonanimal model) HIV reservoir, in tandem with animal model studies to inform on safety, pharmacokinetics, and other parameters important for clinical progression of LRAs (76).

In summary, this study delineates the roles of BET proteins and their BDs in HIV latency and provides a rationale for exploration of IAPi+BETi in animal models of HIV latency. Additionally, detailed mechanistic studies of IAPi+BETi LRA activity indicate full HIV transcriptional elongation, splicing, and associated Tat induction are important barriers to latent HIV protein expression following latency reversal. Development of approaches that increase the rates of full transcriptional elongation and splicing of reactivated proviruses may greatly amplify latency reversal efficacy.

## Methods

### Jurkat latency model screen for IAPi-based LRA combinations.

A Jurkat latency model derived from Jurkat clone E6-1 cells (ATCC, TIB-152), as described in detail elsewhere (4), containing a 1:1:1 ratio of 3 different clones (C16, I15, and N6), which were selected for HIV expression quiescence but inducibility, was used to conduct a targeted screen for combination latency reversal regimens. Each clone has a luciferase reporter in place of *nef*, and the N6 clone has an additional cell-surface murine HSA reporter. Cells were maintained as described previously, and dose-response curves of various LRAs dissolved in DMSO were cross titrated in a 10 × 10 grid against a dose-response curve of IAPi using a D300e Digital Dispenser (Hewlett-Packard). All conditions were normalized to 0.5% (v/v) DMSO. Following incubation for the time periods indicated, the viral luciferase reporter luminescence was measured using Steady-Glo Luciferase (Promega) and an EnVision 2102 Multilabel Plate Reader (PerkinElmer) according to the manufacturer’s instructions. Luminescence values were normalized to percentage of the background luminescent signal from DMSO-treated cells run on each plate.

### Bliss independence analysis of IAPi+BETi synergy.

To assess Bliss independence (25) of the IAPi+BETi combination, DMSO-normalized single agent (IAPi or BETi) and combination values were the converted to a fraction of maximal response E_PMA/i_ (DMSO-normalized RLU values for 10 nM PMA/1 μM ionomycin-treated wells) and assessed for Bliss independence using the equation E_IAPi+BETi_ predicted = (E_IAPi_ + E_BETi_) – (E_IAPi_ × E_BETi_). The E_IAPi+BETi_ predicted value represents the expected value for additivity. The observed value of E_IAPi+BETi_ was subtracted from the predicted value of E_IAPi+BETi_ to yield a Bliss index such that values greater than 0 indicate synergy, values equal to 0 indicate additivity, and values less than 0 indicate antagonism. This analysis was also applied for IAPi+BET protein KOs and primary cell studies of IAPi+BETi.

### Assessment of cell-surface HSA reporter in Jurkat latency model.

The N6 cell line contains heat-stable antigen (HSA) cell-surface reporter within the provirus to facilitate quantitation of the frequency of latently reactivated cells undergoing reactivation. Cells were stimulated for indicated periods of time, washed, and stained with LiveDead Fixable Aqua or Violet (Invitrogen, catalog L34957 and L34955) and rat anti-mouse CD24(HSA)-PE (BD, catalog 553262, clone M1/69) at 4°C for 30 minutes in the dark. Cells were washed twice in PBS/2%FBS, fixed, and analyzed on the BD LSR Fortessa. Analysis of percentage of HSA expression was conducted on live single-cell gates using FlowJo, version 10.6.2.

### scRNA-Seq of N6 Jurkat cells.

N6 cells were treated for 24 hours with DMSO, I-BET151, IAPi, and both I-BET151 and IAPi. Single-cell transcriptomes were generated using the 10× genomics 3′ sequencing method (version 3.1). scRNA-Seq analysis was performed using Cell Ranger (version 6.1.1; 10x Genomics) and Seurat (version 4). The N6 viral genome was determined based on a reference sequence of the parental plasmid NLCH luciferase vector (4). A custom reference genome containing the human (GRCh38p13, UCSC) and N6 provirus (NLCH-Luciferase-mHSA) genome was generated using the Cell Ranger mkref function. Alignment and deconvolution were performed with the Cell Ranger count function. Quality control filtering and secondary analysis were performed in Seurat. Single-cell transcriptome quality was inspected, and 10× barcodes (“cells”) were excluded based on standard quality control metrics of RNA counts and percentage of genes mapping to mitochondrial genomes. The following numbers of single-cell transcriptomes were characterized for each sample before and (after) quality control in Seurat: DMSO, 11394 (10196); IAPi, 10916 (10260); I-BET151, 3723 (3337); IAPi and I-BET151, 8687 (7940). The 4 sample data sets were combined using the merge function and normalized with sctransform to account for differences in read depth with the I-BET151 sample (77). Normalized matrices were examined using UMAP projection, and plots of sctransform normalized expression counts were generated with Seurat visualization functions. Sequencing data are deposited in the NCBI’s Gene Expression Omnibus (GEO GSE196091).

### BET protein polyclonal KO experiments.

Jurkat N6 cells were transduced with sgRNAs targeted against a scramble control, BRD2, BRD3, BRD4 (both isoforms), and BRD4 (long isoform only) and selected in puromycin for approximately 1 week (Supplemental Table 2 and Supplemental Methods). Following selection, KO was verified by immunoblot (Supplemental Methods and Supplemental Figure 4), and the remainder of cells were stimulated with DMSO or IAPi and assessed for HSA expression.

### Assessment of HIV gag RNA induction and cytotoxicity in primary resting CD4^+^ T cells.

PBMCs isolated from aviremic participants on ART were viably thawed. Resting or total CD4^+^ T cells were isolated as indicated and cultured in IMDM supplemented with 10% FBS and 1% penicillin/streptomycin (cIMDM) overnight with abacavir and raltegravir to prevent viral replication from any spontaneously reactivated HIV. The next day, approximately 8 million cells per condition at a density of 1 to 2 × 10^6^ cells/mL were stimulated with indicated concentrations of LRAs or DMSO. Following the indicated incubation times, cells were sampled for analysis of cytotoxicity using acridine orange/propidium iodide (AOPI) staining using the Cellometer (Nexcelom Biosciences) and the CellTiter-Glo Luminescent Cell Viability Assay (Promega, catalog G7570), a measure of total cellular ATP, according to the manufacturer’s instructions. The remainder of the culture was used to prepare 4 to 5 replicates of 1 to 2 × 10^6^ cells per donor per condition for cell-associated HIV *gag* RNA measurements. RNA was extracted using the NucleoMag RNA Kit (Takara Bio, catalog 744350) on an automated KingFisher (Thermo Fisher) platform according to the manufacturer’s instructions. Eluted RNA was diluted 1:3 or 1:4 in molecular grade water, and approximately 100 to 200 ng of RNA per cell replicate was added in triplicate to Fastvirus 1-Step Master Mix (ThermoFisher, catalog 4444434) with primers/probes (final concentration of 900 nM for each primer, 250 nM for each probe) for a conserved region within HIV *gag* (6). Quantities of *gag* defined using a gblock standard curve (Integrated DNA Technologies) were normalized to *TBP* expression, a validated reference gene for lymphocytes and LRA evaluation (78). For experiments with PROTACs, approximately 3 million cells were pelleted for immunoblot confirmation of BET degradation (Supplemental Methods).

### Assessment of HIV p24 induction and viral outgrowth in resting CD4^+^ T cells.

For p24 protein induction, resting CD4^+^ T cells were cultured as described above in the presence of antiretrovirals at a density of approximately 5 million/mL. Following 40 hours of drug stimulation, cell pellets of 4 to 5 million cells per condition and 800 μL of associated culture supernatant were harvested. Cell pellets were lysed and supernatant was inactivated with 1% Triton X-100, then subjected to p24 immunoprecipitation and ultrasensitive digital ELISA p24 measurement as described elsewhere (7). Viral outgrowth assays were conducted and infectious units per million values were determined as described previously, with an overnight drug exposure followed by washout and propagation of reactivated virus (79, 80).

### HIV transcript profiling and p24 protein measurement following IAPi+BETi in total CD4^+^ T cells.

Transcript profiling was conducted as described previously (46). Briefly, approximately 15 million total CD4^+^ T cells per LRA treatment condition were treated for 8 hours with indicated drug concentrations. Following this, approximately 10 million cells were pelleted for analysis of different HIV transcripts using TriReagent RNA extraction and the cDNA synthesis and digital PCR methodologies described in detail elsewhere (46). An average of 5.3 μg of RNA (quantified using Nanodrop UV/Vis Spectrophotometer) was loaded into each cDNA reaction for duplicate digital PCR determinations of absolute copies of read-through, elongated LTR, *pol*, *tat-rev*, polyadenylated, and *nef* transcripts. An additional cDNA reaction for the specific quantification of TAR transcripts was also included, as described (46). The remainder (approximately 5 million) of CD4^+^ T cells were washed extensively and cultured at a density of 5 million cells/mL in cIMDM out to 72 hours in the presence of abacavir and raltegravir with serial sampling of the culture supernatant for ultrasensitive digital ELISA p24 detection, as described previously (7).

### Statistics.

Differential expression analysis for scRNA-Seq data was performed on sctransform normalized values using Wilcoxon’s rank-sum test using the Seurat FindMarkers function with cutoffs for gene features expressed in at least 10% of cells, a log_2_-fold difference of at least 0.25, and Bonferroni’s correction for multiple comparisons based on the total number of genes in the data set. Pairwise comparisons of BET proteins KOs and/or drug treatments to one another or control conditions across different assays were performed using Wilcoxon’s rank-sum tests or Wilcoxon’s matched pairs signed rank tests as appropriate, with FDR corrections such that less than 1 false-positive significant result is estimated to occur for the indicated number of comparisons in each figure. Adjusted *P* values of less than 0.05 were considered significant.

### Study approval.

Study participants were recruited through the UNC Global HIV Prevention and Treatment Clinical Trials Unit and the UNC Center for AIDS Research HIV Clinical Cohort. This study was approved by the Biomedical Institutional Review Board of UNC, and all participants provided written, informed consent prior to participation. Participants were stably suppressed on ART (HIV-1 RNA <50 copies/mL) for at least 6 months prior to enrollment (Supplemental Table 1) and had CD4^+^ T cell counts of 300 cells/μL or more.

## Author contributions

SDF, JJP, AMWT, DI, RMD, NMA, and DMM designed research. SDF, JJP, AMWT, DI, JR, SLMR, KSJ, CS, AS, AE, GS, RF, BA, SG, JLK, GW, EPB, and PZ performed research. IR, RCF, RKP, LIJ, EPB, BDS, and BJH contributed new reagents/analytic tools. SDF, JJP, AMWT, DI, AE, RS, and RMD analyzed data. CB, JDK, and CLG recruited study participants and provided clinical support. SDF, NMA, and DMM wrote the manuscript with input from all authors.

## Supplementary Material

Supplemental data

Supplemental data set 1

## Figures and Tables

**Figure 6:**
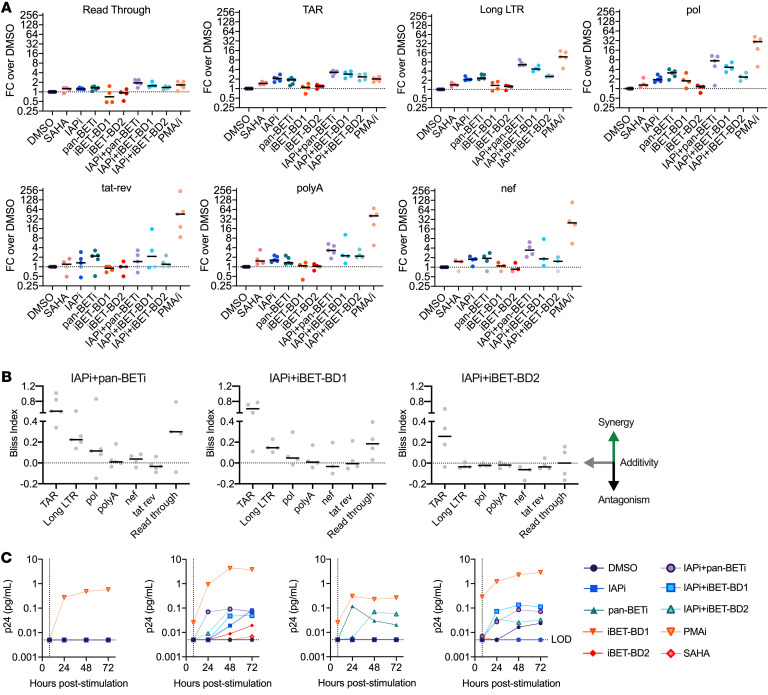
26. **HIV transcript profiling and p24 protein induction following IAPi and/or BETi exposure in primary CD4^+^ T cells from aviremic donors.** (**A**) Fold change values for different HIV transcripts normalized to μg RNA input in total CD4^+^ T cells following 8 hours of LRA stimulation. Each dot represents the average fold change over DMSO-treated cells for an individual donor. Donors (A-2, D-3, H-2, J, K-2) were tested for IAPi/pan-BETi (I-BET151) and single agents; 4 donors were tested for IAPi, pan-, and BD-selective-BETi (D-3, H-2, J, K-2) (Supplemental Table 1). For 1 donor there was a likely PCR amplification failure for the *nef* transcript. In 2 donors, there were insufficient cells to evaluate the HDACi suberoylanilide hydroxamic acid (SAHA) in parallel. Filled symbols represent conditions where there were no detectable transcripts above background and data were left censored at 5 copies/μg RNA input, based on the background digital droplet signal observed in no reverse transcriptase and no template control wells run for each donor on each plate for each primer/probe set. Horizontal bars indicate the median fold change across the donors tested for an indicated transcript/drug condition. (**B**) Bliss synergy indexes for IAPi+BETi drug combinations across different transcripts. Horizontal bars indicate the median index calculated across 4 to 5 donors depending on the transcript/drug condition. (**C**) Ultrasensitive p24 measurements in culture supernatants following 8 hours drug exposure and washout for 4 different donors (D-3, H-2, J, K-2).

## Version 1 04/15/2022

Electronic publication
