# Supplementary material for: Combined noncanonical NF-κB agonism and targeted BET bromodomain inhibition reverse HIV latency ex vivo
Source: J Clin Invest. 2022 Apr 15;132(8):e157281. doi: 10.1172/JCI157281 (PMC9012286; doi:10.1172/JCI157281)
Supplement: Supplemental data [file jci-132-157281-s194.pdf]

Supplementary Information for

Combined non-canonical NF-κB agonism and targeted BET bromodomain inhibition reverses HIV latency ex vivo

Shane D. Falcinelli[1,2], Jackson J. Peterson[1,2], Anne-Marie W. Turner[1,3], David Irlbeck[1,4], Jenna Read[1], Samuel L.M. Raines[1], Katherine S. James[1], Cameron Sutton[1,5], Anthony Sanchez[1], Ann Emery[1,5], Gavin Sampey[1], Robert Ferris[1,4], Brigitte Allard[1], Simon Ghofrani[1], Jennifer Kirchherr[1], Caroline Baker[3], JoAnn D. Kuruc[1,3], Cynthia L. Gay[1,3], Lindsey I. James[1,6], Guoxin Wu[7], Paul Zuck[7], Inmaculada Rioja[8], Rebecca C. Furze[8], Rab K. Prinjha[8], Bonnie Howell[7], Ronald Swanstrom[1,5], Edward P. Browne[2,3], Brian D. Strahl[1,5], Richard M. Dunham[1,4], Nancie M. Archin[1,3], David M. Margolis*[1,2,3]

[1]UNC HIV Cure Center, University of North Carolina, Chapel Hill, NC, 27516, USA.

[2]Dept. of Microbiology and Immunology, University of North Carolina School of Medicine, Chapel Hill, NC, 27516, USA.

[3]Division of Infectious Diseases, Department of Medicine, University of North Carolina, Chapel Hill, NC, USA

[4]HIV Drug Discovery, ViiV Healthcare, Research Triangle Parks, NC, USA

[5]Dept. of Biochemistry and Biophysics, University of North Carolina School of Medicine, Chapel Hill, NC, 27516, USA.

[6]Division of Chemical Biology and Medicinal Chemistry University of North Carolina Eshelman School of Pharmacy, Chapel Hill, NC, 27516, USA.

[7]Department of Infectious Disease, Merck & Co., Inc., Kenilworth, New Jersey, USA.

[8]Immuno-Epigenetics, Immunology Research Unit, GSK Medicines Research Centre, Stevenage, United Kingdom.

*Corresponding author. CB# 7042, Genetic Medicine Building 120 Mason Farm Road Chapel Hill, NC 27599-7042. Email: david_margolis@med.unc.edu Phone: +1 (919) 966-6388

This PDF file includes:

Supplementary Methods
Figures S1 to S11
Tables S1 to S2
Supplementary References

Other supplementary materials for this manuscript include the following:

Datasets S1

Supplementary Methods

Latency Reversal Agents The following compounds in DMSO solvent (except where indicated) were used in this study: IAPi AZD5582 (ChemieTek cat#CT-A5582), Vorinostat (Cayman cat#10009929), Panobinostat (Cayman #13280), Ingenol B (LC Laboratories cat# I-9922), GS-9620 (MedChemExpress cat#HY-15601), GSK343 (Sigma cat# SML0766), GSK3i (UNC Center for Integrative Chemical Biology and Drug Discovery), EED226 (MedChemExpress cat# HY-101117), disulfiram (MedChemExpress HY-B0240), I-BET151 (GlaxoSmithKline and ChemieTek (Cat# CT-BET151), JQ1 (AstaTech, Inc.), iBET-BD1 GSK789 (GlaxoSmithKline), iBET-BD2 GSK046 (GlaxoSmithKline), ZXH 3-26 (Tocris cat# 6713), Phorbol-12-myristate-13-acetate (PMA, Sigma cat#5005820001), ionomycin (Sigma cat#I3909), Remel™ PHA Purified (aqueous) (Thermo cat# R30852801), and recombinant human IL-2 (aqueous) (Peprotech cat#200-02).

BET Protein Polyclonal Lentiviral knockout vectors were constructed in the pHKO23 vector (generous gift, Tom Eisenhaure) which enables expression of both CAS9-P2A-PuroR and a target-specific sgRNA after ligating sgRNA spacer sequences into the tandem BsmbI restriction sites. sgRNA sequences against the indicated target were designed using the BROAD sgDesigner webtool. Complementary oligonucleotides encoding the sgRNA spacer sequences were synthesized (Integrated DNA technologies) with the addition of overhanging 5’ CACCG and ACCC sequences on forward and reverse oligonucleotide respectively. Complementary oligos were phosphorylated by T4 Polynucleotide Kinase at 37°, annealed by slow cooling from 95° C to 20° C, and ligated into HKO-23 vector that had been linearized by BsmbI and dephosphorylated with Calf Intestinal Phosphatase (New England Biolabs). Clonal plasmids were generated by transformation into STBL3 bacteria (Invitrogen) and first screened by digestion with BsmbI and BamHI, where successful clones lack intact BsmbI sites. Sanger sequencing of the sgRNA insert region confirmed successful molecular cloning. CRISPR-CAS9 encoding lentiviral particles were generated by co-transfection of 293T/17 cells (ATCC #CRL-11268) cultured in Dulbecco’s modified eagle medium (DMEM, Gibco) with HKO-23 vectors and the psPAX2 (Addgene #12260) and MD2.G-VSVg (Addgene #12259) packaging vectors using the LT1 transfection reagent (Mirus) at a 3:1 volume:DNA mass ratio. After 24 hours 293T/17 cell media was changed to complete RPMI; 48 hours after transfection lentivirus containing supernatants were harvested by clarifying centrifugation and filtration through 0.45 micron syringe filters. Lentiviral supernatant was concentrated using the LentiX lentiviral concentrator reagent which is added at a 1:4 ratio and enables refrigerated centrifugation at 1600 RCF to concentrate lentiviral particles (Takara). Lentiviral pellets were resuspended at 100x of the original concentration in PBS and stored at -80° C. Transduction of latently infected Jurkat cell lines was conducted as follows: $3x10^5$ cells per transduction condition were resuspended in 90 uL of media containing 4 µg/mL polybrene and 10 µL of concentrated viral particles. Cells were spinoculated for 30 min at 600 RCF in a tabletop centrifuge. Following spinoculation, cells were brought to a volume of 1 mL in media lacking polybrene. For the Jurkat N6 cell line, which is cultured in the presence of the reverse-transcriptase inhibitor Efavirenz, cells were rinsed and plated without Efavirenz 24 hours prior to transduction. Efavirenz was returned to the culture media 48 hours after transduction to prevent HIV viral spread. Transduced cells were incubated for 48 hours and then subjected to 72 hours of selection in the presence of culture media containing 0.6 µg/mL puromycin, with the eradication of untransduced cells cultured in puromycin in parallel to confirm successful transduction and

selection. Transduced cells were then cultured 1-2 days in the absence of puromycin before beginning inhibitor experiments and collection for immunoblot analysis.

Western blots for BET CRISPR/CAS9 knockout or BET PROTAC degradation confirmation PROTAC Western Blot Cells were lysed in a modified RIPA buffer (25 mM Tris-HCl pH 7.5, 150 mM NaCl, 1% Triton X-100, 0.5% sodium deoxycholate, 0.1% SDS, 1X complete protease inhibitor (Roche), 1X HALT Phosphatase inhibitor (ThermoFisher), 1 mM sodium butyrate, and 4 µL/mL Benzonase (Sigma) for 30 minutes on ice and cellular debris pelleted. Recovered supernatants were assayed for protein concentrations by the Detergent Compatible Bradford Assay (Pierce, ThermoFisher) according to manufacturer's instructions. Protein lysates were heated at 70deg C with 4x LDS sample buffer (NuPAGE, ThermoFisher) and DTT (NuPAGE 10x reducing agent, ThermoFisher)before being separated by 3-8% Tris-Acetate or 4-20% Tris-Glycine acrylamide gel electrophoresis (Bio-Rad) and transferred onto Immun-Blot PVDF membranes via semi-dry transfer using the Trans-Blot Turbo System (Bio-Rad) or the iBlot2 system (ThermoFisher). Membranes were blocked with tris-buffered saline (TBS) and 5% milk for at least 30 minutes. The following antibodies were used: BRD4L (A700-005, Bethyl, Clone BL-151-6F11), BRD4L/S (ab128874, Abcam, Clone EPR5150(2)), BRD2 (ab139690, Abcam, Clone EPR7642), BRD3 (ab50818, Abcam, Clone 2088C3a), GAPDH (ab83956, Abcam, polyclonal), LaminB1 (ab133741, Abcam, Clone EPR8985(B)), αTubulin (ab7291, Abcam, Clone DM1A), total histone H3 (ab1791, Abcam, polyclonal), and B-Actin (ab49900, Abcam, Clone AC-15). Antibodies were diluted to appropriate concentration in TBS with 0.1% Tween-20 (TBST) with 5% milk and incubated overnight at 4°C. Membranes were washed 3X in TBST and then incubated with appropriate HRP-conjugated secondary antibody (Life Technologies) at 1:10,000 dilution in TBST/milk for 1hr at room temperature. Membranes were washed 3X with TBST, then developed using SuperSignal West Pico Plus ECL (ThermoFisher). Blots were imaged using the Bio-Rad Versadoc imager and analyzed using Image Lab software. Membranes were stripped with Restore PLUS Western Blot Stripping Buffer (Thermo Scientific) and re-probed as necessary. Densitometric quantification was performed in Bio-Rad's Image Lab software.

Cell fractionation and western blots of BD-selective BETi in Jurkat N6 cells. Cells were lysed in modified CSK buffer (10 mM Pipes, NaOH pH 7.0, 340 mM sucrose, 100 mM NaCl, 3 mM $MgCl_2$, 1 mM EGTA, 1mM DTT, 1× complete protease inhibitor (Roche), 1× PhosStop phosphatase inhibitor (Roche), 10% Triton X-100) for 10 minutes on ice. Half of the lysate was saved as total fraction and Pierce Universal Nuclease was added (ThermoFisher). The remaining lysate was pelleted and recovered supernatants were saved as soluble fraction. The remaining pellet was washed with CSK buffer (-Triton), then resuspended in CSK buffer (+Triton, + nuclease) and saved as chromatin fraction. All lysates were intubated on ice for 20 min. Chromatin fraction lysate was sonicated with SONIFIER Cell Disruptor 350 (Branson) for 5x 1s pulses at 20% duty cycle and output control of 4. Proteins were separated by 8% Bis-Tris acrylamide gel and transferred onto Immun-Blot PVDF membranes via semi-dry transfer using Hoefer Semi-Phor transfer unit. Primary antibodies are diluted to appropriate concentration in tris-buffered saline (TBS) with 0.5% Tween-20 (TBST) with 5% milk and incubated overnight at 4 °C. Membranes were washed 3X in TBST and then incubated with appropriate HRP-conjugated secondary antibody (GE Healthcare Biosciences) at 1:10 000 (Rabbit) or 1:5000 (mouse) dilution in TBST/milk for 1 h at room temperature. Membranes were washed 3× with TBST, then developed using Amersham ECL Prime (GE Life Sciences). Blots were imaged using the BioRad ChemiDoc MP imager and analyzed using Image Lab software. Antibodies: BRD4 (ab128874 , Abcam, Clone EPR5150(2)); Alpha Tubulin (T9026, Sigma Aldrich, Clone DM1A).

Assessment of IAPi-inducible host gene induction in CD4 T cells Total CD4 T cells were isolated and stimulated as described in the Figure 6 legend and main methods section. For four of the donors in Figure 6, cDNA was diluted used as an input to measure gene expression of *BIRC3*, *NFKB2, GAPDH*, and *TBP* across drug treatment conditions. Fold change values over DMSO were calculated for each drug treatment, with normalization to *TBP* expression (1). Taqman assays for *NFKB2* (Hs00174517_m1) and *BIRC3* (Hs00985031_g1), Integrated DNA

Technology pre-designed assay for spliced *GAPDH* (Hs.PT.39a.22214836), and previously described assay for *TBP* (2) were used.

Evaluation of IAPi/BETi in 2D10 and 2B5 cell lines Previously described 2D10 and 2B5 cell lines with GFP proviral reporters were viably thawed and cultured in RPMI1640 + glutamine with 10% heat-inactivated FBS and 1% PenStrep (3). Cells were plated in drug-printed plates (D300e Digital Dispenser (Hewlett-Packard)) at a density of approximately 0.2-0.3x10^6 cells/mL for 48 hours. After 48 hours, cells were stained with a Live/Dead viability stain and washed/fixed for flow cytometric analysis on the IntelliCyt iQue Screener PLUS or BD LSR Fortessa with the BD High Throughput Sampler. FCS files were analyzed for GFP expression in FlowJo version 10.6.2 with a live single cell gate.

Measurement of nascent translation HIV seronegative PBMCs were sourced from New York Blood Center. CD4 T cells were isolated from buffy PBMCs using StemCell CD4 isolation kit (cat#17952). Cells were cultured at approximately 2x10^6 cells/mL in a 24 well-plate and exposed to the indicated concentrations of drugs. Following the incubation period, cells were fixed and stained using the Protein Synthesis Assay Kit (Cayman Chemical cat#601100) according to the manufacturer's instructions. Analysis of nascent translation signal using 6-carboxyfluorescein (FAM)-labeled O-Propargyl-puromycin (OPP) was conducted on single cell gates using FlowJo version 10.6.2. Median fluorescence intensity values were normalized to DMSO signal for analysis.

Nuclear fractionation experiments PBMCs isolated from aviremic participants on ART were viably thawed and cultured in IMDM supplemented with 10% FBS and 1% PenStrep overnight with abacavir and raltegravir to prevent viral replication from spontaneously reactivated HIV. CD4 T cells were isolated using StemCell CD4 isolation kit (cat#17952), and then stimulated with indicated concentrations of compounds for 24 hours (4 million CD4 per condition) in the presence of antiretrovirals prior to pelleting at 2000 rpm x 5 minutes. Cells were immediately subject to cytoplasmic lysis on ice per the manufacturer's instructions using the Active Motif RNA subcellular isolation kit (cat #25501). RNA from each fraction was treated with PerfeCTa ® DNase I (RNase-free) (cat#95150-100) per manufacturer's instructions prior to use in cDNA reaction with qScript master mix (Quanta Bio cat#95048-025). cDNA was assayed for HIV *gag* expression (4) using Bio-Rad digital PCR according to the manufacturer's instructions in quadruplicate with normalization to RNA input into the cDNA reaction. Fractionation control digital droplet PCR reactions were also prepared for RPS14 transcripts (5) (Taqman assay ID: Hs00852033_g1), spliced *GAPDH* transcripts (IDT predesigned assay: Hs.PT.39a.22214836), and unspliced *GAPDH* transcripts (from IDT; forward primer: CCACCAACTGCTTAGCACC, FAM probe AGGCTCCCACCTTTCTCATCCAAGACTGGCTC, and reverse primer CTCCCCACCTTGAAAGGAAAT) (6). Fractionation efficiency was also confirmed using n = 2 western blots of CD4 T cells following isolation of fractions but before RNA extraction. For western blots, nuclear pellets were lysed in a modified RIPA buffer (25 mM Tris pH 8, 150 mM NaCl, 1% NP-40, 1% Sodium Deoxycholate, 0.1% SDS, 1x complete protease inhibitor (Roche), and 1 uL Pierce universal nuclease per 100 uL buffer) for 30 minutes on ice and cellular debris pelleted. Concentration of resulting supernatants from nuclear lysis and cytosolic fractions were measured by the Detergent Compatible Bradford Assay (Pierce, ThermoFisher) according to manufacturer's instructions. Samples were then analyzed as described in the Western blot methods above for fractionation efficiency. Antibodies: LaminB1 (ab133741, Abcam, Clone EPR8985(B)), αTubulin (ab7291, Abcam, Clone DM1A), total histone H3 (ab1791, Abcam, polyclonal).

Analysis of HIV reads in 10X Genomics data from Jurkat N6 cells Total HIV transcripts described in Figure S1 were quantified using Seurat according to methods described in main manuscript. Molecular details on HIV transcript distribution (Figure S10) were assessed as follows. For each sample, raw reads were aligned to a combined reference of the human genome (hg38) and the HIV sequence in N6 cells using the 10x Genomics Cellranger 6.1.1 pipeline. From the aligned .bam output files, the HIV reads were extracted using bamtools (version 2.4.0)

to extract reads aligning to HIV (7). The 10x single cell 3' sequencing platform is intended to capture poly-A reads and is therefore generally restricted to 3' sequencing; however, we found evidence that mispriming occurs across the entire A rich genome of HIV. Information from multiple HIV reads with the same UMI was combined and reads with poor quality scores were removed. All HIV reads were then sorted and combined by cell barcode and UMI, with cell barcodes filtered to specifically examine whitelisted cell barcodes identified by Cellranger. Using an in-house Python script/Jupyter notebook, HIV reads were identified as specific transcript types based on their location and splicing information. Sequencing data is deposited in the NIH Gene Expression Omnibus (GEO) Accession# GSE196091.

Assessment of polyadenylated HIV *gag* and *NFKB2* transcripts following IAPi+BETi treatment. 10-20M ART-suppressed donor total CD4 T cells were stimulated with DMSO or 100 nM AZD5582 and 1000 nM I-BET151 for 8 hours. Total RNA was isolated previously described (8). Approximately 1 µg of total RNA was reserved for total RNA cDNA synthesis, and the remainder (up to 9.5 µg) was added to washed Dynabeads™ Oligo(dT)25 beads (ThermoFisher cat# 61002) and mRNA was isolated according to the manufacturer's "purify mRNA from total RNA" instructions. Total RNA and mRNA were quantified using the Qubit™ RNA high sensitivity (HS) kit (ThermoFisher cat#Q32852). Approximately 1 µg of total RNA and 100 ng of mRNA underwent cDNA synthesis as described previously (8). cDNA was subject to digital PCR for HIV *gag, TBP*, and *NFKB2* on the Bio-Rad QX200 platform, and *TBP*-normalized fold change over DMSO values across total and mRNA fractions were calculated for HIV *gag* and *NFKB2* (Taqman assay Hs00174517_m1) (1, 2, 4).

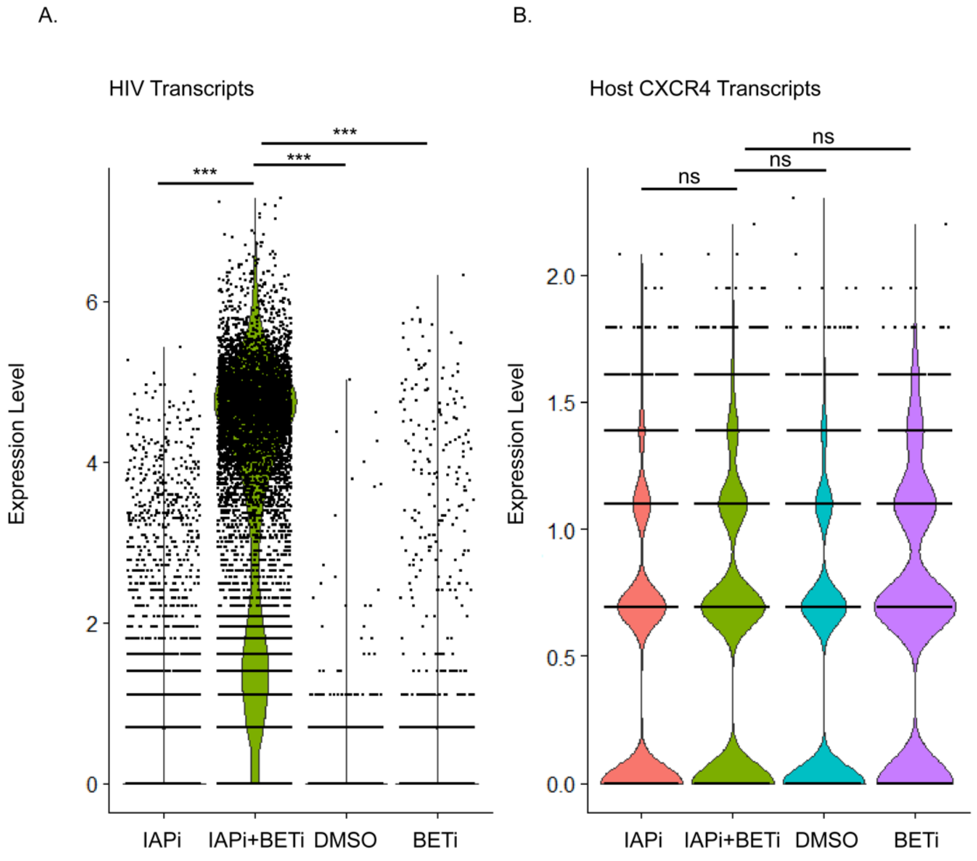

Figure S1. Absence of synergistic CXCR4 transcript induction upon addition of I-BET151 to IAPi. Violin plot of HIV transcript and CXCR4 transcript expression in Jurkat N6 cells following the indicated drug treatments (100 nM IAPi AZD5582, 1 μM BETi I-BET151 or combination thereof). Y-axes indicates $\log_2$ transcripts detected per-cell after sctransform normalization function in Seurat. Wilcoxon rank-sum tests were performed to compare expression levels of HIV transcripts and CXCR4 transcripts in the IAPi+BETi condition to other drug treatment conditions with Bonferroni correction for multiple comparisons. *** = adjusted p value < 0.001, ns = not significant.

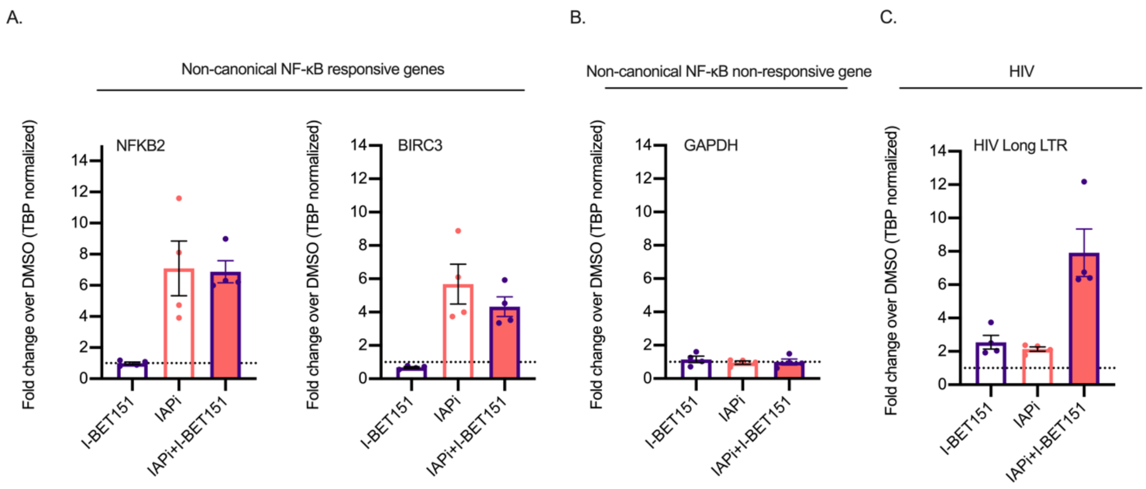

Figure S2. Absence of synergistic transcript induction for IAPi-responsive host genes upon addition of I-BET151. CD4 T cells from HIV seropositive ART-suppressed donors were stimulated for 8 hours with the indicated LRAs (100 nM IAPi AZD5582 or 1 μM I-BET151). This data is derived from the same cDNA used in the main Figure 6. RNA was extracted and subject to cDNA synthesis and digital PCR using primer/probes sets for the indicated targets with normalization to the reference gene *TBP*. (A) non-canonical NF-κB responsive genes, (B) control gene non-responsive to non-canonical NF-κB signaling and (C) HIV elongated LTR transcript. Each dot represents the mean induction over DMSO for an individual donor (n=4). Error bars represent standard error of the mean.

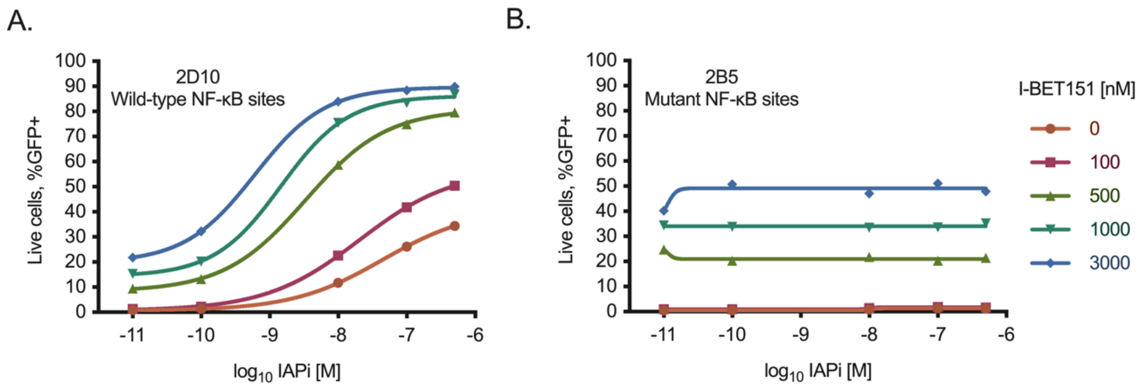

Figure S3. Combination IAPi/BETi activity is dependent on NF-κB sites in the HIV promoter. (A) 2D10 (H13L *tat,* wild-type NF-κB sites) and (B) 2B5 (H13L *tat,* mutant NF-κB sites) Jurkat cells were stimulated for 48 hours with the indicated LRA exposures (3). IAPi (AZD5582) single agent activity and combination IAPi/BETi latency reversal activity (GFP proviral reporter expression) was abrogated in the presence of mutant NF-κB sites in the HIV promoter (2B5 cells). Conditions without IAPi treatment were plotted as 1×10^{-11} M for visualization on the $\log_{10}$ x axis. Dose response curves were generated using GraphPad Prism 9 using a log(agonist) vs. response variable slope four parameter curve fit. Representative of two independent experiments.

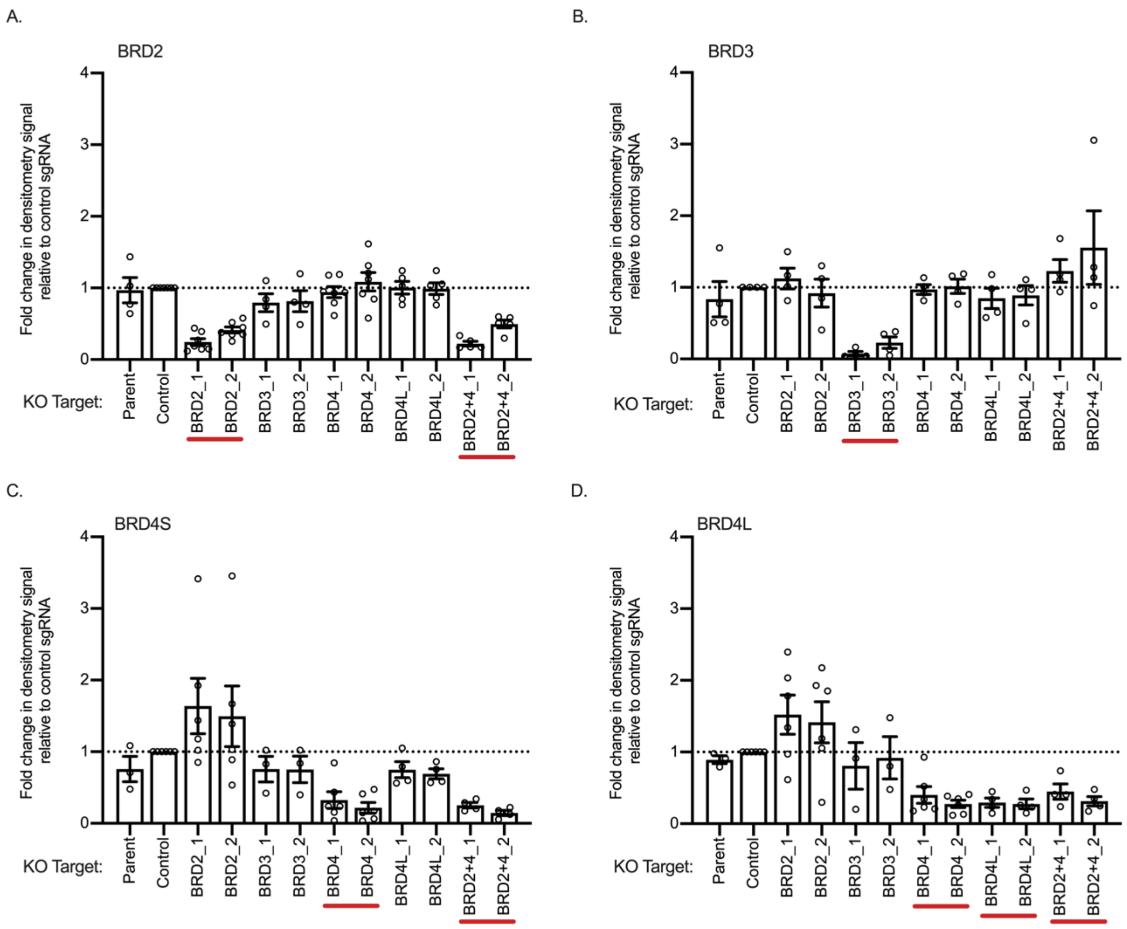

Figure S4. Densitometric quantification of CRISPR/CAS9 knockdown of BET proteins in Jurkat N6 cells. Densitometric signal for each BET protein band was normalized to that of β-actin loading control. Y axes represent the fold change in β-actin normalized densitometric signal relative to cells transduced with an empty vector control for (A) BRD2 bands, (B) BRD3 bands, (C) BRD4S bands, and (D) BRD4L bands. Red lines indicate the BET proteins targeted with sgRNAs for each plot. BRDX_1 and BRDX_2 designations indicate different sgRNAs for each target (i.e. BRD2_1 is 1 of 2 sgRNAs used to target BRD2) (Table S2). Data derived from 7 independent experiments, with each target quantified in at least n=4 experiments. Error bars represent standard error of the mean. Associated with main Figure 3.

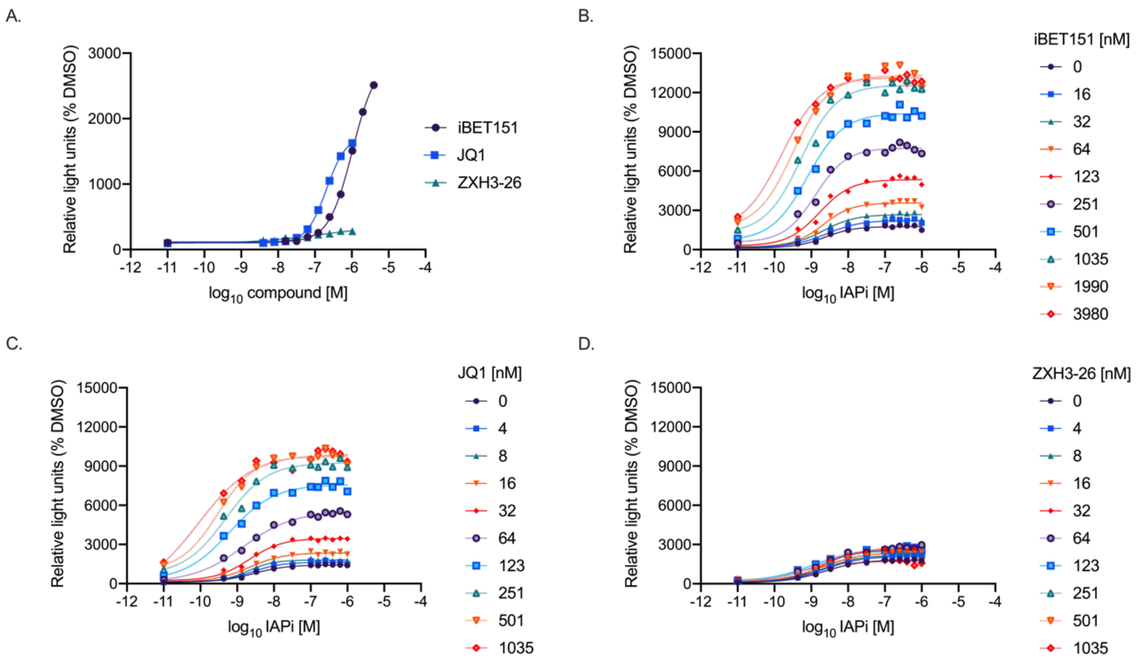

Figure S5. Reduced combination activity with IAPi (AZD5582) when BET-targeted PROTAC ZXH3-26 is used versus the parent JQ1 BET inhibitor in the triple Jurkat model. Dose-response curves for (A) single agents (B) IAPi/ IBET151, (C) IAPi/parent JQ1 inhibitor, and (D) BET-targeted PROTAC ZXH3-26. Luciferase signal from 24 hr LRA exposure in the triple Jurkat model represented as a percentage of DMSO signal. Conditions without IAPi treatment were plotted as 1×10^{-11} M for visualization on the $\log_{10}$ x axis. Dose response curves were generated using GraphPad Prism 9 using a log(agonist) vs. response variable slope four parameter curve fit. Representative of three independent experiments.

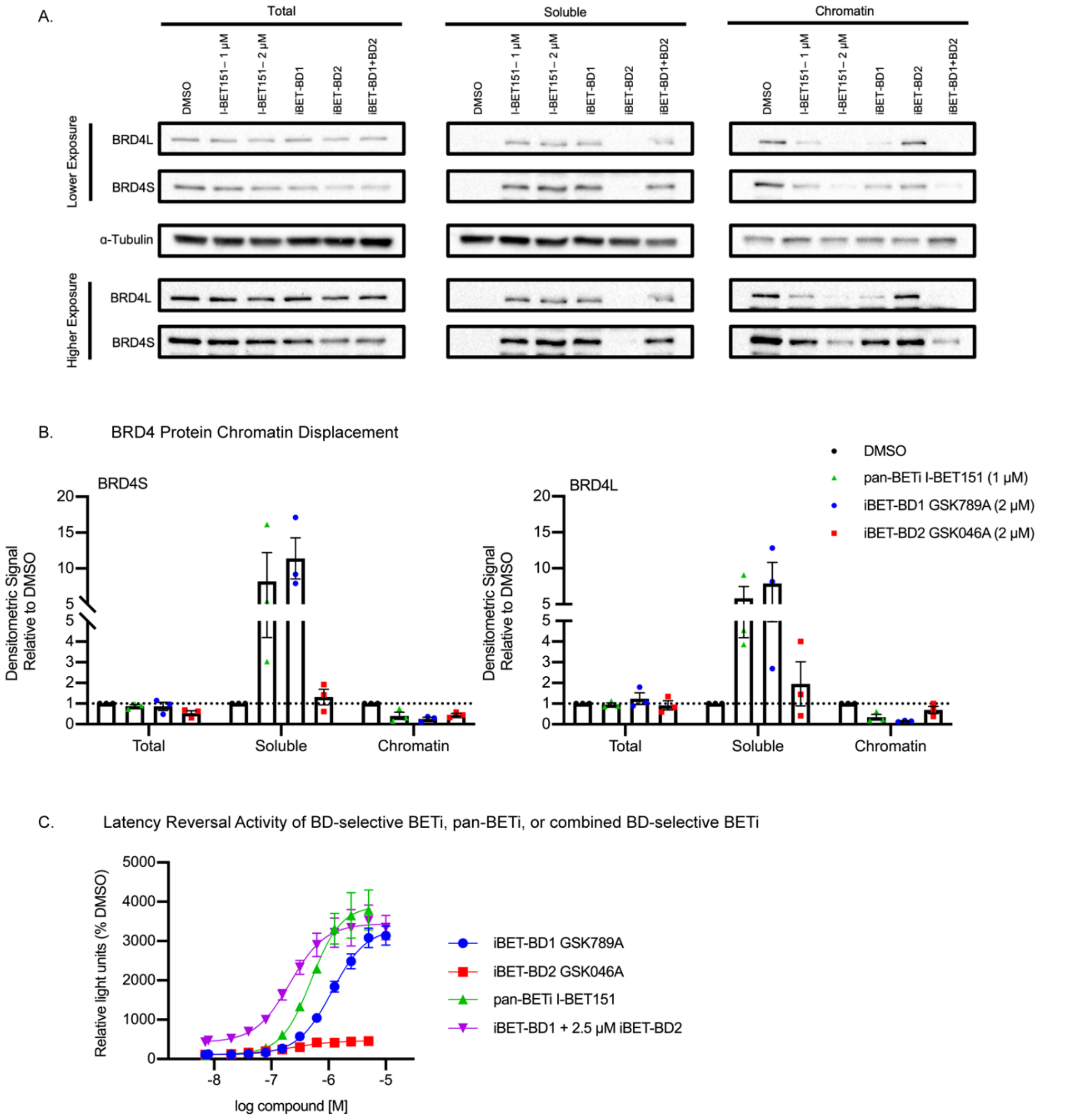

Figure S6. Efficient BET protein displacement from chromatin with pan- and BD1-selective BETi but not BD2 BETi in Jurkat cells. (A) Representative western blot of cellular fractions from Jurkat N6 cells treated overnight with the pan-BETi I-BET151 (1 or 2 µM), iBET-BD1 (2 µM), iBET-BD2 (2 µM) or iBET-BD1+iBET-BD2 (both 2 µM) BETi, fractionated into total, soluble (including cytoplasmic) and chromatin partitions, and assessed for BRD4 levels. Top BRD4L/BRD4S image represents shorter exposure (used for quantitation) and bottom image represents longer exposure. Representative of three independent experiments. 2 µM I-BET151 and iBET-BD1+iBET-BD2 conditions were performed in one experiment. Similar results were obtained with an 8 h drug exposure (n=1, data not shown). (B) pan-BETi and iBET BD1 efficiently displacement BRD4 isoforms from chromatin. Some reduction in BRD4S signal from the chromatin fraction was observed for iBET-BD2, but this was also observed in the total input fraction. Densitometric quantification of all n=3 independent experiments for 1 µM pan-BETi I-BET151, 2 µM iBET-BD1, and 2 µM iBET-BD2. Concentrations of I-BET151, iBET-BD1, and iBET-BD2 were chosen to target approximately 85% inhibition of the respective bromodomain(s) based on time-resolved fluorescence energy transfer and other assays (data not shown). Error bars represent standard error of the mean. (C) Combined BD-selective BETi result in similar maximal activity to pan-BETi. Dose response curves for pan-BETi and BD-selective BETi in the triple Jurkat model as in main Figure 5, but also displaying dose response curve for iBET+BD1 in combination with a >EC_{90} fixed dose of iBET-BD2 (2.5 µM). Error bars represent standard error of the mean across n=3 independent experiments.

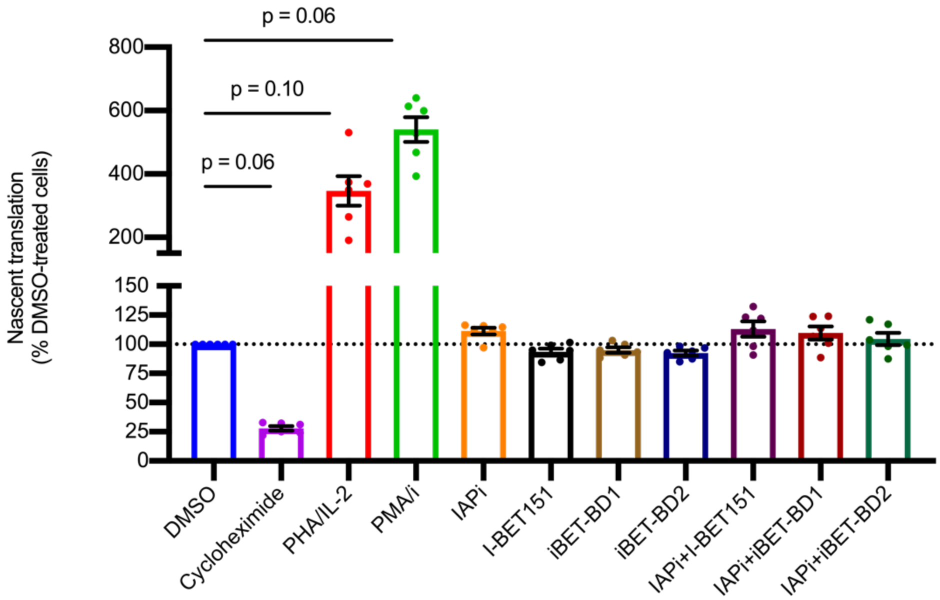

Figure S7. Minimal impact of BETi on nascent cellular translation. (A) Nascent cellular translation levels measured by FAM-labeled O-Propargyl-puromycin (OPP) incorporation into ribosomes in primary CD4 T lymphocytes derived from n=6 HIV seronegative donors following 24-hour exposure to mitogens, IAPi (100 nM AZD5582), BETi LRAs: I-BET151 (1 μM), iBET-BD1 (2 μM), iBET-BD2 (2 μM), and combinations. Each dot represents nascent translation level relative to DMSO-treated cells in CD4 T cells from an individual donor. Error bars represent standard error of the mean. FDR-adjusted p values less than or equal to 0.1 are shown (FDR (5%) corrected Friedman test with pairwise comparisons of each condition to DMSO).

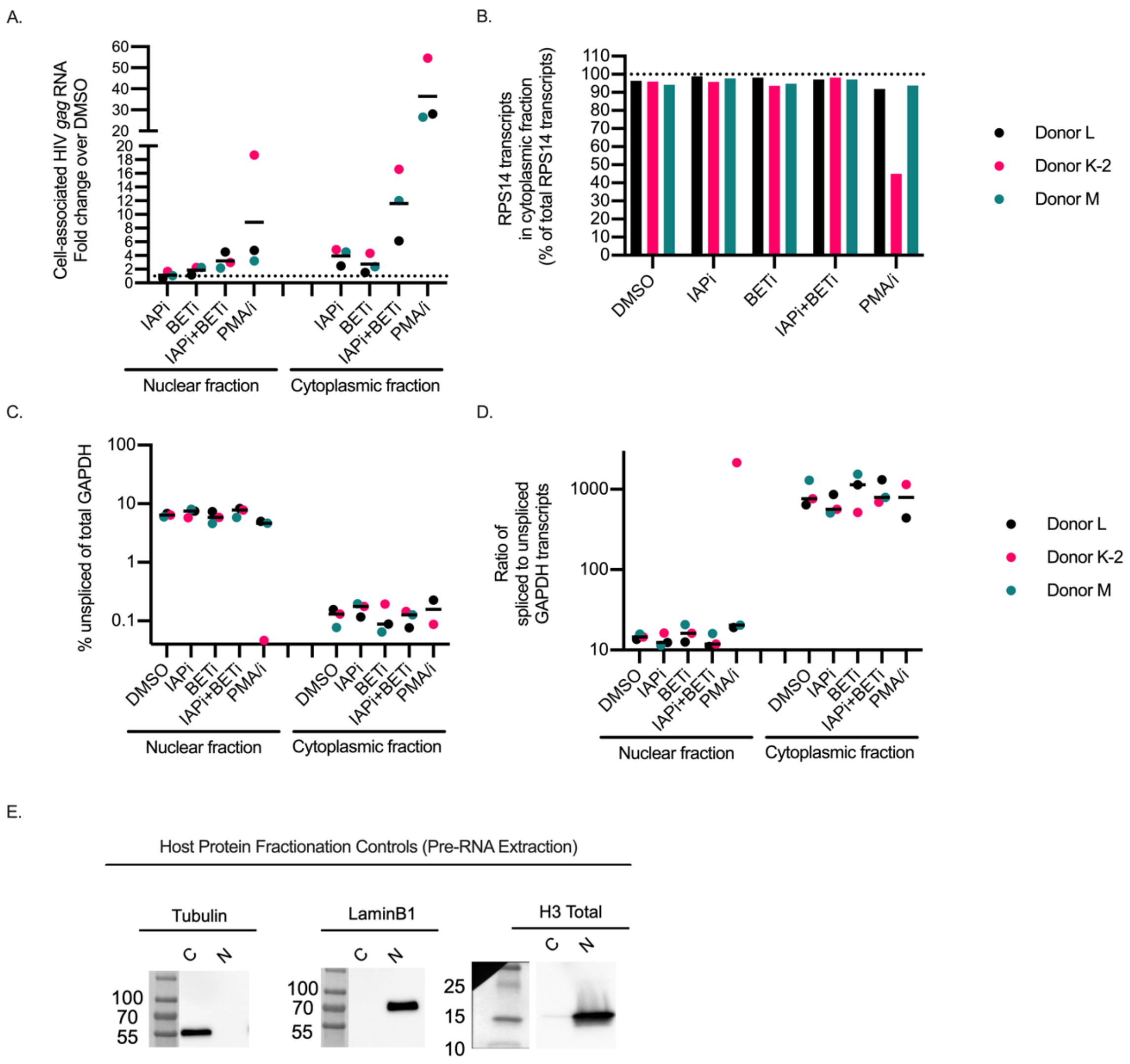

Figure S8. Nuclear export of HIV *gag* RNA following IAPi+BETi treatment in CD4 T cells from ART-suppressed donors. (A) Cell-associated HIV *gag* RNA inductions relative to DMSO for nuclear and cytoplasmic RNA fractions following 24 h exposure to IAPi (100 nM AZD5582) and/or BETi (1 μM I-BET151) treatment in n=3 ART-suppressed donors. Fold change values were calculated using the mean of four ddPCR replicates for each treatment condition/donor. (B) Host *RPS14* RNA fractionation control shows expected enrichment of *RPS14* RNA in cytoplasmic fractions (5); y axis represents cytoplasmic *RPS14* transcripts as a percentage of total *RPS14* RNA (sum of cytoplasmic copies of transcript/μg RNA and nuclear RNA copies of transcript/μg RNA). (C,D) Assessment of spliced and unspliced *GAPDH* transcripts within each nuclear or cytoplasmic fraction for each drug treatment across the n = 3 donors tested. (C) Consistent enrichment (across donors and drug treatments) of unspliced *GAPDH* transcripts in the nuclear fraction and (D) spliced *GAPDH* transcripts in the cytoplasmic fraction (6, 9, 10). (E) Western blot confirmation of efficient cellular fractionation at the protein level prior to RNA extraction in CD4 T cells. Western blot is representative of two independent experiments.

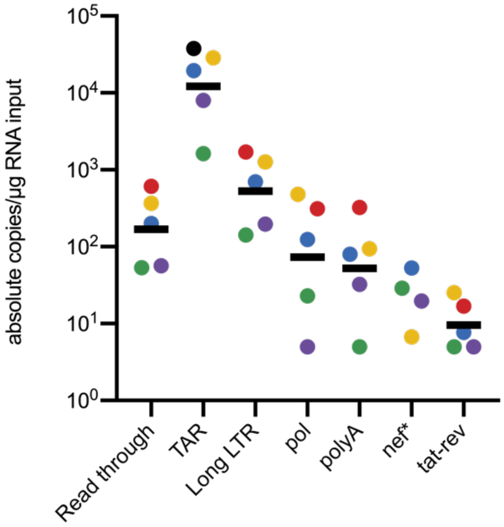

Figure S9. Baseline absolute frequency of different HIV transcripts in DMSO-treated total CD4 T cells from n=5 ART-suppressed donors. Each dot represents the average of 2 technical replicates from a different donor: A-2, D-3, H-2, J, K-2. Horizontal black bars indicate geometric mean of transcript level across all donors. No geometric mean bar is provided for *nef* because one donor (red dots) had a likely PCR amplification failure and frequency of *nef* transcripts could not be determined. Absolute frequencies of different transcripts are similar to that originally reported by Yukl and colleagues (8).

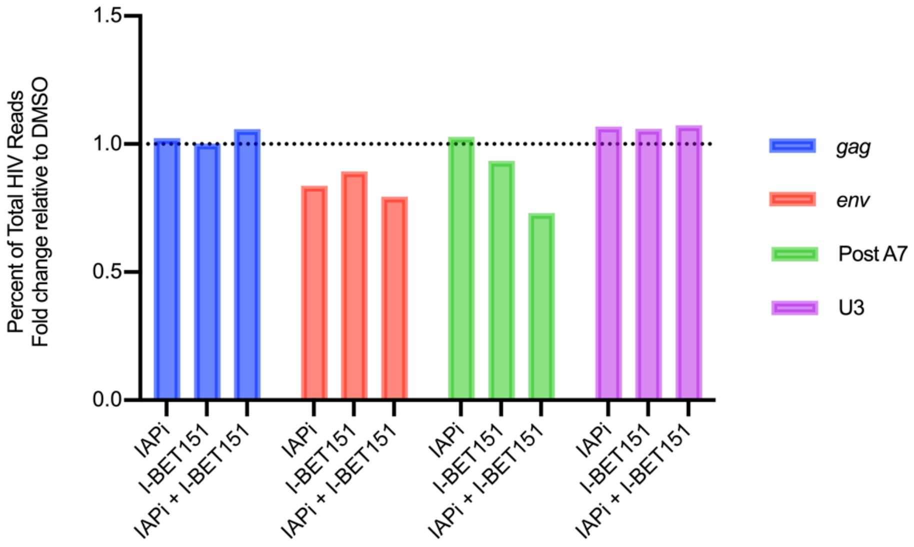

Figure. S10. Limited evidence for impaired transcriptional elongation in the Jurkat N6 latency model following 24 hour exposure to IAPi (100 nM AZD5582) and or pan-BETi (I-BET151 1 μM). Percentage of HIV reads of total HIV reads from scRNAseq data were calculated for for *gag, env,* Post A7, and U3 regions for each drug treatment condition. Y axis indicates the percentage of total reads as a fold change relative to DMSO-treated cells. Percentages are based on a total of 449296 reads for *gag*, 119118 reads for *env*, 14674 reads for post A7, and 94039 reads for U3.

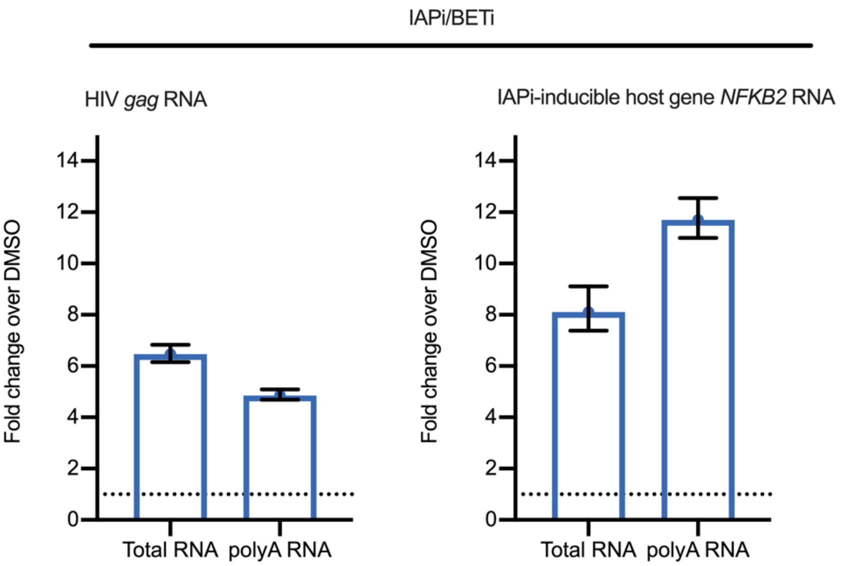

Figure S11. Comparison of IAPi (100 nM AZD5582) + pan-BETi (I-BET151 1 μM) fold induction relative to DMSO-treated cells across total and polyA RNA fractions for HIV *gag* RNA (left) versus *NFKB2* RNA (right), an IAP-inducible host gene following an 8 h drug exposure. Representative data for donor K-2 are shown; decreased polyadenylated *gag* relative to total *gag* transcripts was observed for all 3 donors tested. Error bars represent 95% confidence intervals for the fold change estimate derived from total error estimates in the Quantasoft analysis software.

Table S1. ART-suppressed HIV seropositive donor clinical information.

Donor ID	Most Recent CD4 Count	HIV Status at Treatment Initiation	Years on ART	Years of ART-suppression	ART at Leuk	ART at Leuk	Age at Leuk	Sex at Birth	Race	Ethnicity
A-1	1034	Chronic	33.9	11.2	Biktarvy	B/FTC/TAF	59	M	White	Non-Hispanic
A-2	653	Chronic	34.6	12.0	Biktarvy	B/FTC/TAF	60	M	White	Non-Hispanic
B	1045	Acute	1.0	0.8	Triumeq	3TC/ABC/DTG	33	M	Black	Non-Hispanic
C	528	Chronic	9.4	9.3	Biktarvy	B/FTC/TAF	52	M	Black	Non-Hispanic
D-1	514	Acute	2.1	1.9	Triumeq	3TC/ABC/DTG	26	M	Black	Non-Hispanic
D-2	394	Acute	4.6	4.4	Biktarvy	B/FTC/TAF	28	M	Black	Non-Hispanic
D-3	496	Acute	5.0	4.8	Biktarvy	B/FTC/TAF	29	M	Black	Non-Hispanic
E-1	1120	Chronic	5.5	5.4	Odefsey	TAF/FTC/RPV	34	M	Black	Non-Hispanic
E-2	1120	Chronic	5.7	5.6	Odefsey	TAF/FTC/RPV	34	M	Black	Non-Hispanic
F	977	Chronic	4.6	4.3	Biktarvy	B/FTC/TAF	27	M	White	Non-Hispanic
G	1169	Chronic	9.1	8.9	Triumeq	3TC/ABC/DTG	48	F	Black	Non-Hispanic
H-1	916	Acute	2.1	2.0	Triumeq	3TC/ABC/DTG	25	M	Black	Non-Hispanic
H-2	916	Acute	2.8	2.7	Triumeq	3TC/ABC/DTG	26	M	Black	Non-Hispanic
I	928	Chronic	20.1	4.7	Biktarvy	B/FTC/TAF	41	M	Black	Non-Hispanic
J	654	Chronic	11.6	10.2	Genvoya	EVG/COBI/FTC /TAF	60	F	Black	Non-Hispanic
K-1	631	Chronic	8.4	2.2	Biktarvy	B/FTC/TAF	52	M	White	Non-Hispanic
K-2	538	Chronic	8.8	2.5	Biktarvy	B/FTC/TAF	52	M	White	Non-Hispanic
L	525	Chronic	8.3	7.4	Biktarvy	B/FTC/TAF	47	M	White	Non-Hispanic
M	788	Chronic	11.2	6.7	Descovy, Tivicay	FTC/3TC, DTG	42	M	Other	Hispanic

Table S2. sgRNA protospacer sequences for CRISPR/CAS9 knockout experiments.

sgRNA	Sequence
sgBRD2_1	CCAGCTGCAATACCTACACA
sgBRD2_2	ACAGTGGTAGGTATCTCAGG
sgBRD3_1	CGAGAGTACCCAGACGCACA
sgBRD3_2	CACAACCACTCCCACGACGT
sgBRD4_1	TCTTCCTCCGACTCATACGT
sgBRD4_2	AGTCGAACTGTCACTGTCCG
sgBRD4L_1	TTCACCCCGGCACCACAAGT
sgBRD4L_2	GCAGAACGCACCGCCACCAG

SI References

1. Ceriani C, Streeter GS, Lemu KJ, James KS, Ghofrani S, Allard B, et al. Defining stable reference genes in HIV latency reversal experiments. *J Virol.* 2021.
2. Falcinelli SD, Shook-Sa BE, Dewey MG, Sridhar S, Read J, Kirchherr J, et al. Impact of biological sex on immune activation and frequency of the latent HIV reservoir during suppressive antiretroviral therapy. *J Infect Dis.* 2020.
3. Pearson R, Kim YK, Hokello J, Lassen K, Friedman J, Tyagi M, et al. Epigenetic silencing of human immunodeficiency virus (HIV) transcription by formation of restrictive chromatin structures at the viral long terminal repeat drives the progressive entry of HIV into latency. *J Virol.* 2008;82(24):12291-303.
4. Malnati MS, Scarlatti G, Gatto F, Salvatori F, Cassina G, Rutigliano T, et al. A universal real-time PCR assay for the quantification of group-M HIV-1 proviral load. *Nat Protoc.* 2008;3(7):1240-8.
5. Chow EY, Zhang J, Qin H, and Chan TF. Characterization of Hepatocellular Carcinoma Cell Lines Using a Fractionation-Then-Sequencing Approach Reveals Nuclear-Enriched HCC-Associated lncRNAs. *Front Genet.* 2019;10:1081.
6. Blissenbach M, Grewe B, Hoffmann B, Brandt S, and Uberla K. Nuclear RNA export and packaging functions of HIV-1 Rev revisited. *J Virol.* 2010;84(13):6598-604.
7. Barnett DW, Garrison EK, Quinlan AR, Stromberg MP, and Marth GT. BamTools: a C++ API and toolkit for analyzing and managing BAM files. *Bioinformatics.* 2011;27(12):1691-2.
8. Yukl SA, Kaiser P, Kim P, Telwatte S, Joshi SK, Vu M, et al. HIV latency in isolated patient CD4(+) T cells may be due to blocks in HIV transcriptional elongation, completion, and splicing. *Sci Transl Med.* 2018;10(430).
9. Lassen KG, Ramyar KX, Bailey JR, Zhou Y, and Siliciano RF. Nuclear retention of multiply spliced HIV-1 RNA in resting CD4+ T cells. *PLoS Pathog.* 2006;2(7):e68.
10. Imamichi H, Dewar RL, Adelsberger JW, Rehm CA, O'Doherty U, Paxinos EE, et al. Defective HIV-1 proviruses produce novel protein-coding RNA species in HIV-infected patients on combination antiretroviral therapy. *Proc Natl Acad Sci U S A.* 2016;113(31):8783-8.
